# Long‐term repopulation of aged bone marrow stem cells using young Sca‐1 cells promotes aged heart rejuvenation

**DOI:** 10.1111/acel.13026

**Published:** 2019-08-05

**Authors:** Jiao Li, Shu‐Hong Li, Jun Dong, Faisal J. Alibhai, Chongyu Zhang, Zheng‐Bo Shao, Hui‐Fang Song, Sheng He, Wen‐Juan Yin, Jun Wu, Richard D. Weisel, Shi‐Ming Liu, Ren‐Ke Li

**Affiliations:** ^1^ Guangdong Key Laboratory of Vascular Diseases, State Key Laboratory of Respiratory Disease, The Second Affiliated Hospital, Guangzhou Institute of Cardiovascular Disease Guangzhou Medical University Guangzhou China; ^2^ Division of Cardiovascular Surgery, Toronto General Hospital Research Institute University Health Network Toronto ON Canada; ^3^ Division of Cardiac Surgery, Department of Surgery University of Toronto Toronto ON Canada

**Keywords:** aging, heart, reconstitution, rejuvenation, Sca‐1, stem cells

## Abstract

Reduced quantity and quality of stem cells in aged individuals hinders cardiac repair and regeneration after injury. We used young bone marrow (BM) stem cell antigen 1 (Sca‐1) cells to reconstitute aged BM and rejuvenate the aged heart, and examined the underlying molecular mechanisms. BM Sca‐1^+^ or Sca‐1^−^ cells from young (2–3 months) or aged (18–19 months) GFP transgenic mice were transplanted into lethally irradiated aged mice to generate 4 groups of chimeras: young Sca‐1^+^, young Sca‐1^−^, old Sca‐1^+^, and old Sca‐1^−^. Four months later, expression of rejuvenation‐related genes (Bmi1, Cbx8, PNUTS, Sirt1, Sirt2, Sirt6) and proteins (CDK2, CDK4) was increased along with telomerase activity and telomerase‐related protein (DNA‐PKcs, TRF‐2) expression, whereas expression of senescence‐related genes (p16^INK4a^, P19^ARF^, p27^Kip1^) and proteins (p16^INK4a^, p27^Kip1^) was decreased in Sca‐1^+^ chimeric hearts, especially in the young group. Host cardiac endothelial cells (GFP^−^CD31^+^) but not cardiomyocytes were the primary cell type rejuvenated by young Sca‐1^+^ cells as shown by improved proliferation, migration, and tubular formation abilities*.* C‐X‐C chemokine CXCL12 was the factor most highly expressed in homed donor BM (GFP^+^) cells isolated from young Sca‐1^+^ chimeric hearts. Protein expression of Cxcr4, phospho‐Akt, and phospho‐FoxO3a in endothelial cells derived from the aged chimeric heart was increased, especially in the young Sca‐1^+^ group. Reconstitution of aged BM with young Sca‐1^+^ cells resulted in effective homing of functional stem cells in the aged heart. These young, regenerative stem cells promoted aged heart rejuvenation through activation of the Cxcl12/Cxcr4 pathway of cardiac endothelial cells.

## 1 INTRODUCTION

The idea that age itself is one of the major risk factors for the development of cardiovascular disease (CVD) has motivated interest in the field of cardiac aging. Growing clinical and experimental evidence shows that the aging process promotes structural and functional remodeling of the heart, even in the absence of overt CVD (Keller & Howlett, 2016). The current understanding of the mechanisms implicated in age‐related CVD includes telomere shortening and cellular senescence (Chen et al., 2014), mitochondrial oxidative stress (Kornfeld et al., 2015), as well as genomic instability and chromatin modifications (Lord & Ashworth, 2012). An increase in senescent cells within the vascular wall and heart contributes to the structural and functional decline of the cardiovascular system with age (Huang, Alhenc Gelas, & Osborne‐Pellegrin, 1998). Critical aspects associated with cellular senescence include age‐dependent defects in adrenergic signaling and calcium handling which dampen mechanical efficiency and electrophysiological properties, increasing the risk of arrhythmias. Mitochondrial overproduction of reactive oxygen species (ROS) ultimately leads to the formation of highly reactive superoxide or H_2_O_2_, the accumulation and diffusion of which fosters cellular senescence, DNA mutations, inflammation, and the activation of multiple cell death pathways (Camici, Savarese, Akhmedov, & Luscher, 2015). It has been suggested that sporadic genomic mutations accumulated across the lifespan represent a major underpinning for the development of CVD (Shah & Mahmoudi, 2015). These findings, coupled with the growing aged population worldwide, highlight the urgency of understanding how aging promotes CVD in order to develop new treatment strategies.

Aging impairs the functioning of endogenous stem/progenitor cells, including cardiac progenitor cells (Capogrossi, 2004; Cho, Sieburg, & Muller‐Sieburg, 2008; Liang, Van Zant, & Szilvassy, 2005; Sudo, Ema, Morita, & Nakauchi, 2000). Recent data obtained from hematopoietic stem cells (HSCs) indicate that by the age of 70, clonal diversity has collapsed into quasi‐monotypic hematopoiesis which may influence stem cell competency during ischemia or infarction (Goodell & Rando, 2015). Several studies have documented a reduced number of bone marrow (BM)‐derived cells and circulating angiogenic cells in aged patients, as well as in patients with modifiable CV risk factors (Fadini, Losordo, & Dimmeler, 2012). Features of senescence, such as telomere shortening, genomic instability, subsequent cell cycle arrest, and enhanced cell apoptosis, accompany this reduced cell number (Williamson, Stringer, & Alexander, 2012).

Previously, we evaluated the importance of the function of the cardiac‐resident BM‐derived progenitor cell pool on cardiac recovery after injury in aged animals (Li et al., 2013). A BM transplantation model was developed which employed old C57BL/6 mice aged 20–22 months (old recipients) that were lethally irradiated (10.5 Gy) and then immediately received an infusion (through the tail vein) of fresh BM cells (5 × 10^6^) from C57BL/6‐Tg‐GFP mice aged 2–3 months (young marrow), generating young chimeras or aged 20–22 months (old marrow), generating old chimeras. At steady state, these young BM cells were able to stably integrate into the aged myocardium within areas with a high density of fibronectin near other non‐BM cells. These cardiac‐resident BM‐derived progenitor cells were characterized as positive for myeloid and mesenchymal markers (CD34, c‐Kit, CD45, Tie2, CD44), but negative for cardiac lineage markers (von Willebrand factor, vWF for endothelial cells, smooth muscle actin, SMA for smooth muscle cells, and sarcomeric α‐actinin for cardiomyocytes). Furthermore, we were able to create a unique mouse model with young cardiac‐resident BM cells in old animals, with old peripheral BM cells. To establish this model, after recovery from primary BM reconstitution, young and old chimeric mice underwent a second lethal irradiation followed by an infusion of BM cells from old female donors. During the second irradiation, the heart was protected with a lead shield carefully positioned on the chest, which prevented the radiation‐induced loss of cardiac‐resident BM‐derived progenitor cells introduced into the heart during the first reconstitution but eliminated the peripheral BM cells, which were subsequently replaced with old BM cells in the second reconstitution. This new cardiac‐restricted chimeric protocol allowed us to isolate the origin and capability of these cardiac‐resident BM‐derived progenitor cells and demonstrate for the first time that their functional capacity before injury determined the extent of ventricular functional restoration after injury by paracrine mechanisms. This minority cardiac cell population elicited its effects through paracrine‐directed improvement in survival, proliferation, and neovascularization. Subsequently, we found that stem cell antigen 1 (Sca‐1) cells were the key BM cell type involved in the rejuvenation of the aged heart (Li et al., 2017). We isolated Sca‐1^+^ or Sca‐1^−^ cells from the BM of young donor mice and infused them into lethally irradiated old recipients to generate Sca‐1^+^ or Sca‐1^−^ chimeras, respectively. At homeostasis, these BM Sca1^+^ cells maintained the monocyte/progenitor cell pool in aged heart. After myocardial infarction (MI), these homed BM Sca‐1^+^ cells stimulated proliferation of donor and host progenitor cells in the aged heart which was associated with better restoration of cardiac function. We also evaluated Sca‐1^+^ cell differentiation from 14 days up to 4 months after MI and found that myocardial injury did not initiate their vascular or cardiac differentiation. We further demonstrated that these Sca‐1^+^ subset of young cells improved healing of the aged heart by stimulating cell proliferation through activation of the PDGFRβ‐Akt/p27^Kip1^ signaling pathway. However, the mechanisms and the exact mediators underlying BM Sca‐1^+^ cell‐mediated cardiac rejuvenation of the aged heart remain elusive. In addition, the specific cell type in the aged heart that was rejuvenated by BM Sca‐1^+^ cells has not been identified.

Chronological aging is characterized by biomarkers of cellular senescence, including the upregulation of gene products implicated in growth arrest, the downregulation of rejuvenation‐related genes, and attenuation of telomerase activity (Costantino, Paneni, & Cosentino, 2016). To confirm these phenotypic changes at the organ and cellular level, in the present study, we first compared the expression of senescence‐related and rejuvenation‐related genes in young and old wild‐type mouse hearts. Next, we investigated the possible cross‐talk between BM Sca‐1^+^ cells and the cardiac aging process using in vitro and in vivo experimental models, including reconstitution of aged mice with BM Sca‐1^+^ or Sca‐1^−^ cells from young or aged GFP^+^ transgenic mice. The effects of Sca‐1^+^ cells on rejuvenation of the aged heart before and after MI were investigated as well as the underlying molecular mechanisms involved. Host cardiac endothelial cells (GFP^−^CD31^+^) were identified as the primary cell type rejuvenated by young BM Sca‐1^+^ cells. We demonstrate that homed young BM Sca‐1^+^ cells improved aged recipient cardiac endothelial function and promoted heart rejuvenation through activation of the Cxcl12/Cxcr4 pathway.

## 2 RESULTS

### 2.1 Heart senescence increased with aging

**Figure 1:**
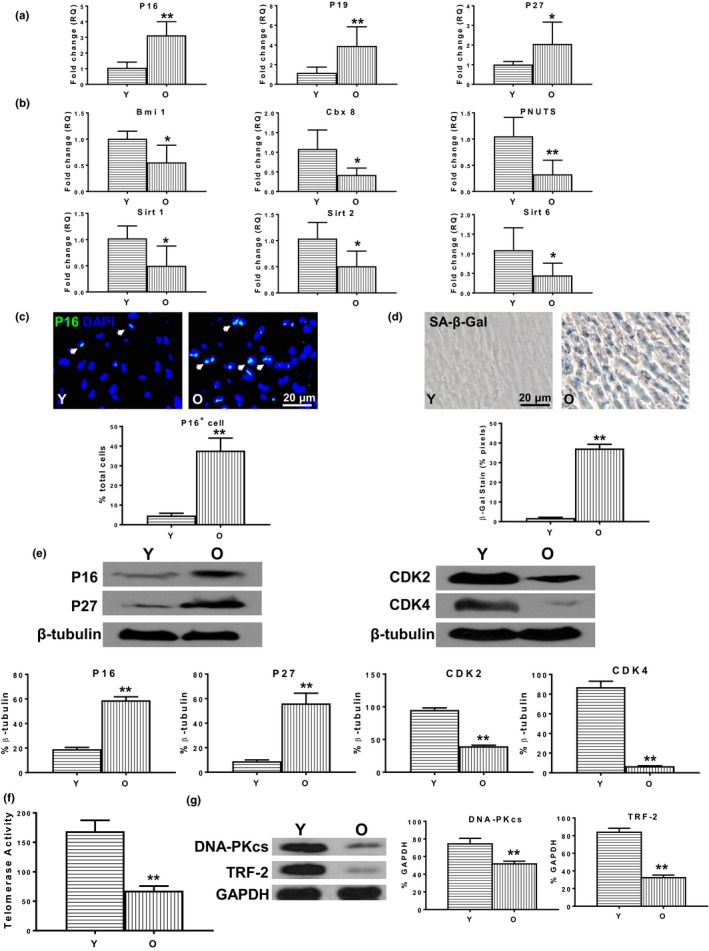
Heart senescence increased with aging. The phenotypic changes in cellular senescence were compared in the hearts of young (Y, 2–3 months) and old (O, 22–23 months) wild‐type (C57BL/6) mice. The expression of (a) senescence‐related genes p16^INK4a^, p19^ARF^, and p27^Kip1^ and (b) rejuvenation‐related genes Bmi1, Cbx8, PNUTS, Sirt1, Sirt2, and Sirt6 in O and Y mouse hearts. (c) More p16^INK4a+^ cells were found in O compared with Y mouse hearts. (d) More senescence‐associated beta galactosidase (SA‐β‐gal)‐positive cells were found in O compared with Y mouse hearts. (e) The protein expression of p16^INK4a^ and p27^Kip1^ and cyclin‐dependent kinase 2 (CDK2) and CDK4 in O and Y mouse hearts. (f) Telomerase activity and (g) telomerase‐related protein (DNA damage kinases [DNA‐PKcs], telomeric repeat binding factor 2 [TRF‐2]) expression were decreased in O compared with Y mouse hearts. *n* = 6/group; *p < .05; **p < .01

To confirm cellular senescence at the organ level, we compared the expression of senescence‐related and rejuvenation‐related genes in young and old wild‐type mouse hearts without infarction. The expression of senescence‐related genes was significantly increased (Figure 1a), and the expression of rejuvenation‐related genes was decreased (Figure 1b) in old compared with young mouse hearts. Immunostaining revealed more p16^INK4a^‐positive cells in old than young mouse hearts (Figure 1c). Senescence‐associated beta galactosidase (SA‐β‐gal) staining revealed more positive cells in old than young mouse hearts (Figure 1d). Western blots confirmed increased expression of senescence‐related proteins p16^INK4a^ and p27^Kip1^ in old relative to young hearts and decreased protein expression of cyclin‐dependent kinase 2 (CDK2) and CDK4 which are downstream genes regulated by p16^INK4a^ and p27^Kip1^, respectively (Figure 1e). On the other hand, telomerase activity (Figure 1f), telomerase‐related protein DNA damage kinases (DNA‐PKcs) expression, and telomeric repeat binding factor 2 (TRF‐2) expression were decreased in old compared to young hearts (Figure 1g). Chronological aging coincided with cardiac cellular senescence which was detected by biomarkers at the organ level.

### 2.2 Cardiac endothelial cells most susceptible to senescence during aging

**Figure 2:**
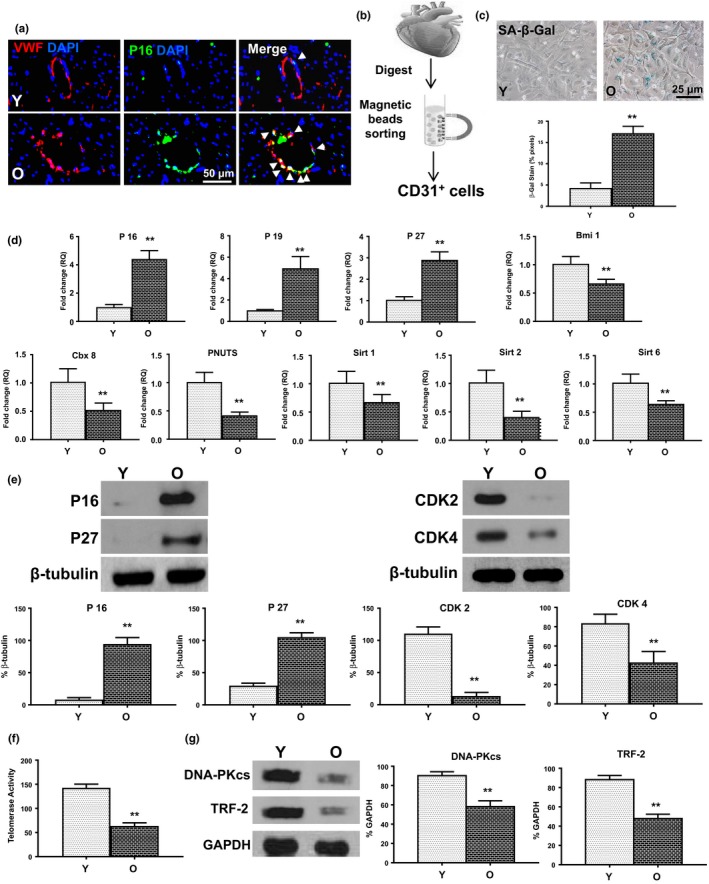
Cardiac endothelial cells most susceptible to senescence during aging. (a) More p16^INK4a^ and Von Willebrand factor (VWF) double‐positive cells (White arrows) located at the inner lumen side of blood vessels in O mouse hearts. (b) CD31^+^ endothelial cells were isolated from Y and O wild‐type mouse hearts. The senescence‐associated beta galactosidase activity (SA‐β‐gal, c) was compared in O and Y cardiac endothelial cells. (d) The mRNA expression of p16^INK4a^, p19^ARF^, and p27^Kip1^ and Bmi1, Cbx8, PNUTS, Sirt1, Sirt2, and Sirt6 was compared in O and Y cardiac endothelial cells. (e) The protein expression of p16^INK4a^, p27^Kip1^, CDK2, and CDK4 was compared in O and Y cardiac endothelial cells. (f) Telomerase activity and (g) telomerase‐related protein (DNA‐PKcs, TRF‐2) expression were compared in O and Y cardiac endothelial cells. *n* = 6/group; **p < .01

To verify whether organ senescence involved cellular senescence of all cell types or a specific cell type, we first conducted immunostaining and found more p16^INK4a^‐positive cells located on the inner lumen side of blood vessels which were also positive for cardiac endothelial marker Von Willebrand factor (VWF^+^) in aged mouse hearts (Figure 2a). Next, we isolated cardiomyocytes (Figure S1A), endothelial cells (Figure 2b), cardiac fibroblasts (Figure S2A), and smooth muscle cells (Figure S3A) for characterization of the senescent phenotype in individual cell types. More SA‐β‐gal positive cells were found in old than young mouse cardiac endothelial cells (Figure 2c). As shown in Figure 2d, the expression of senescence‐related genes was increased and the expression of rejuvenation‐related genes was decreased in old compared with young mouse cardiac endothelial cells. Consistent with these findings, protein expression of p16^INK4a^ and p27^Kip1^ was increased, whereas protein expression of CDK2 and CDK4 was decreased in old compared with young mouse cardiac endothelial cells (Figure 2e). In addition, both telomerase activity (Figure 2f) and telomerase‐related protein expression (Figure 2g) were decreased in aged cardiac endothelial cells. Interestingly, the senescent phenotype observed in the aged cardiac endothelial cells did not appear in other aged cardiac cell types. There was no significant difference between young and old cardiomyocytes (Figure S1B,C), fibroblasts (Figure S2B,C), or vascular smooth muscle cells (Figure S3B,C) with regard to senescence‐ or rejuvenation‐related gene expression. These findings indicate that cardiac endothelial cells are more susceptible to senescence during the aging process of the mouse heart.

### 2.3 Young BM Sca‐1 cells decreased senescence and improved cellular function of aged recipient cardiac endothelial cells

**Figure 3:**
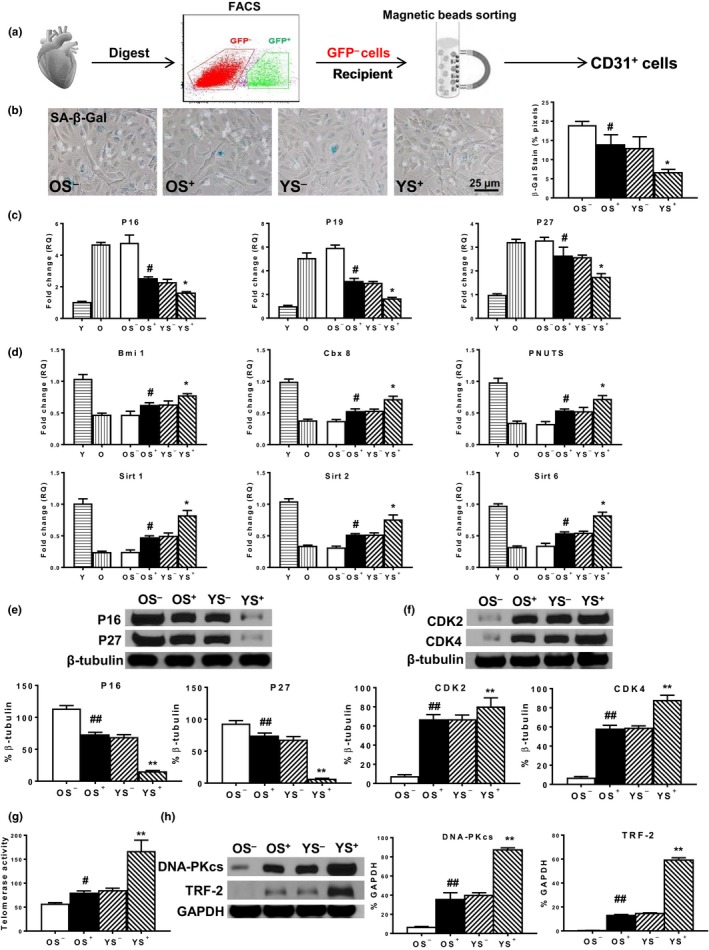
Young BM Sca‐1 cells decreased recipient cardiac endothelial cell senescence after BM reconstitution. BM Sca‐1^+^ or Sca‐1^−^ cells (2 × 10^6^) from young (Y, 2–3 months) or old (O, 18–19 months) GFP transgenic mice were transplanted into lethally irradiated (9.5 Gy) O mice to generate 4 groups of chimeras: Y Sca‐1^+^ (YS^+^), Y Sca‐1^−^ (YS^−^), O Sca‐1^+^ (OS^+^), and O Sca‐1^−^ (OS^−^), respectively. (a) Four months after BM reconstitution, recipient cardiac endothelial cells were isolated from reconstituted mouse hearts using FACS and immunomagnetic bead sorting for GFP^−^CD31^+^ cells. The senescence‐associated beta galactosidase activity (SA‐β‐gal, b) was compared in recipient cardiac endothelial cells among the 4 chimeric groups. The gene expression of (c) p16^INK4a^, p19^ARF^, and p27^Kip1^ and (d) Bmi1, Cbx8, PNUTS, Sirt1, Sirt2, and Sirt6 was compared among the Y and O and the 4 chimeric groups. The protein expression of (e) p16^INK4a^ and p27^Kip1^ and (f) CDK2 and CDK4 was compared among the four chimeric groups. (g) Telomerase activity and (h) telomerase‐related protein (DNA‐PKcs, TRF‐2) expression were compared among the 4 chimeric groups. *n* = 6/group; **p < .01 YS^+^ vs. YS^−^, OS^+^ *,* or OS^−^; *p < .05 YS^+^ vs. YS^−^, OS^+^ *,* or OS^−^; ^##^ *p* < .01 OS^+^ vs. OS^−^, #*p* < .05 OS^+^ vs. OS^−^

Next, young or old BM Sca‐1^+^ or Sca‐1^−^ cells were used to reconstitute old recipients. At 4 month after reconstitution, the young Sca‐1^+^ subset had more uniformly replaced the stem and progenitor cells in the BM, blood, and heart of aged recipients than the other three groups. The number of GFP^+^ cells in the BM, blood, and heart was significantly higher in the BM Sca‐1^+^ compared with the Sca‐1^−^ reconstituted groups, especially in the YS^+^ group (Figure S4). Four months after BM reconstitution, recipient cardiac endothelial cells were isolated from reconstituted mouse hearts using fluorescence‐activated cell sorting (FACS) and subsequent immunomagnetic bead sorting for GFP^−^CD31^+^ cells (Figure 3a). Staining of SA‐β‐gal revealed fewer positive cells in the cardiac endothelial cells of Sca‐1^+^ than Sca‐1^−^ BM reconstituted mice, with the lowest number in the YS^+^ group (Figure 3b). The expression profiles of senescence‐ and rejuvenation‐related genes were assessed by RT‐qPCR. Recipient cardiac endothelial cells from old Sca‐1^−^ chimeras (OS^−^) had the highest gene expression of p16^INK4a^, p19^ARF^, and p27^Kip1^ (Figure 3b). BM reconstitution with old Sca‐1^+^ (OS^+^) or young Sca‐1^−^ (YS^−^) cells effectively decreased expression of these senescence‐related genes. However, BM reconstitution with young BM Sca‐1^+^ (YS^+^) cells yielded the lowest expression (Figure 3c). Conversely, recipient cardiac endothelial cells from the OS^−^ group had the lowest gene expression of rejuvenation‐related genes (Figure 3d). BM reconstitution with old Sca‐1^+^ (OS^+^) or young Sca‐1^−^ (YS^−^) cells effectively increased expression of these rejuvenation‐related genes. However, BM reconstitution with young BM Sca‐1^+^ (YS^+^) cells yielded the highest expression (Figure 3d). Consistent with these findings, the protein expression of p16^INK4a^ and p27^Kip1^ was highest in the OS^−^ group, was comparably lower in the OS^+^ and YS^−^ groups, and was the lowest in the YS^+^ group (Figure 3e). In contrast, the protein expression of CDK2 and CDK4 in recipient cardiac endothelial cells showed the opposite pattern (Figure 3f). Telomerase activity (Figure 3g) and telomerase‐related protein (Figure 3h) expression were lowest in the recipient cardiac endothelial cells of the OS^−^ group, comparably higher in the OS^+^ and YS^−^ groups, and highest in the YS^+^ group. These findings suggest that BM Sca‐1 cells effectively decreased aged recipient endothelial senescence.

To determine whether BM Sca‐1 cells not only decreased cell senescence, but also improved aged endothelial cell function, recipient cardiac endothelial cells (GFP^−^CD31^+^) were isolated from the reconstituted heart and cultured in vitro. The proliferation (Figure S5A), migration (Figure S5B), and tubular formation (Figure S5C) abilities of recipient cardiac endothelial cells were examined. As shown in Figure S5A, the recipient cardiac endothelial cells from the OS^−^ group had the lowest proliferative ability (BrdU^+^ 5‐bromo‐2'‐deoxyuridine] cells). BM reconstitution with old Sca‐1^+^ (OS^+^) or young Sca‐1^−^ (YS^−^) cells effectively increased the number of proliferative recipient cardiac endothelial cells, but the effect was greatest in the YS^+^ group. A similar pattern was found for migration (Figure S5B) and tubular formation (Figure S5C). The ratio of phospho‐eNOS/total eNOS protein, which is the major mediator of endothelial function, was also increased in the recipient cardiac endothelial cells from the OS^+^ and YS^−^ groups relative to the OS^−^ group with the highest ratio found in the YS^+^ group (Figure S5D). These data confirm that BM Sca‐1 cells not only decreased cell senescence, but also improved the function of aged recipient endothelial cells.

### 2.4 Young BM Sca‐1 cells decreased global senescence of aged recipient hearts

**Figure 4:**
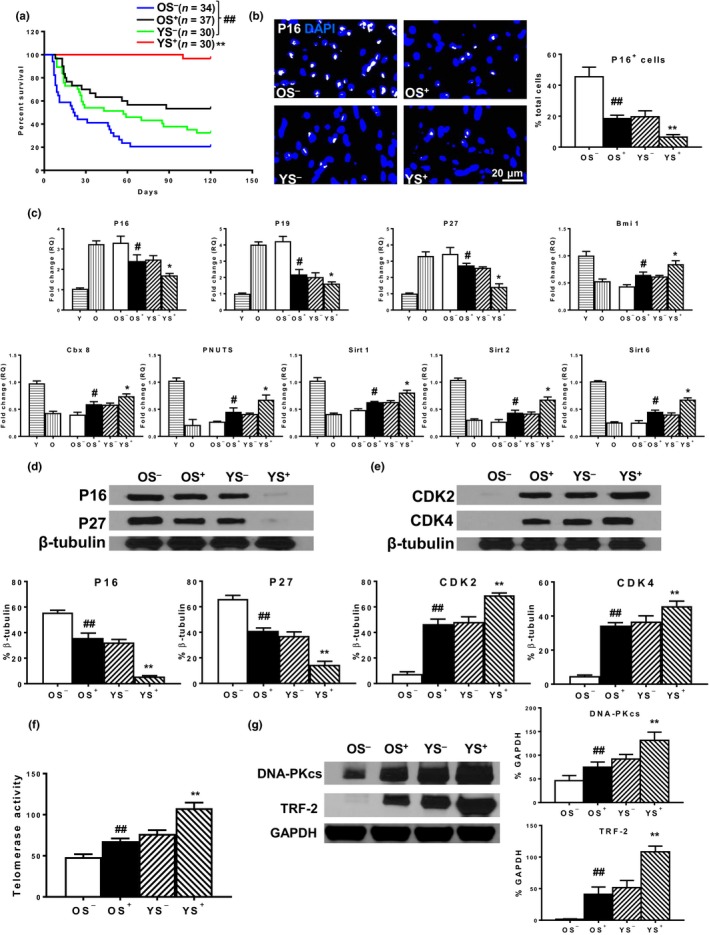
Young BM Sca‐1 cells decreased global senescence of aged recipient hearts. (a) Survival rate of the 4 chimeric groups 4 months after BM reconstitution. (b) p16^INK4a+^ cells and (c) gene expression of p16^INK4a^, p19^ARF^, p27^Kip1^ Bmi1, Cbx8, PNUTS, Sirt1, Sirt2, and Sirt6 among the Y, O, and the 4 chimeric groups. Protein expression of (d) p16^INK4a^ and p27^Kip1^ and (e) CDK2 and CDK4. (f) Telomerase activity and (g) telomerase‐related protein (DNA‐PKcs, TRF‐2) in the 4 chimeric hearts. *n* = 6/group; **p < .01 YS^+^ vs. other group; ^##^ *p* < .01 OS^+^ vs. OS^−^; #*p* < .05 OS^+^ vs. OS^−^

To determine whether by decreasing recipient endothelial cell senescence, BM Sca‐1 cells were capable of attenuating global senescence in aged recipient hearts, animal survival and a global senescent phenotype were examined in the four chimeric groups. Four months after BM reconstitution, the YS^+^ chimeras had the highest survival rate, followed by the YS^−^, OS^+^, and OS^−^ groups (Figure 4a). Immunostaining with p16^INK4a^ to identify senescent cells showed that OS^−^ chimeras had the highest number of p16^INK4a+^ cells followed by the YS^−^, OS^+^, and YS^+^ groups (Figure 4b). OS^−^ chimeric hearts had the highest expression of senescence‐related genes (Figure 4c). BM reconstitution with old Sca‐1^+^ (OS^+^) or young Sca‐1^−^ (YS^−^) cells effectively decreased the expression of p16^INK4a^, p19^ARF^, and p27^Kip1^; however, the YS^+^ group had the lowest expression of these genes (Figure 4c). In contrast, the opposite pattern was observed for rejuvenation‐related gene expression in chimeric hearts (Figure 4c). Consistent with these findings, the protein expression of p16^INK4a^ and p27^Kip1^ was highest in the OS^−^ group, comparably lower in the OS^+^ and YS^−^ groups, and lowest in the YS^+^ group (Figure 4d). Conversely, the opposite pattern was observed for protein expression of CDK2 and CDK4 (Figure 4e). Telomerase activity (Figure 4f) and telomerase‐related protein expression (Figure 4g) were lowest in the recipient hearts of the OS^−^ group, comparably higher in the OS^+^ and YS^−^ groups, and highest in the YS^+^ group. All of this evidence suggests that BM Sca‐1 cells attenuated global senescence in aged hearts by decreasing endothelial senescence and improving endothelial function.

### 2.5 Young BM Sca‐1 cells decreased senescence of aged recipient cardiac endothelial cells through Cxcl12/Cxcr4 pathway

**Figure 5:**
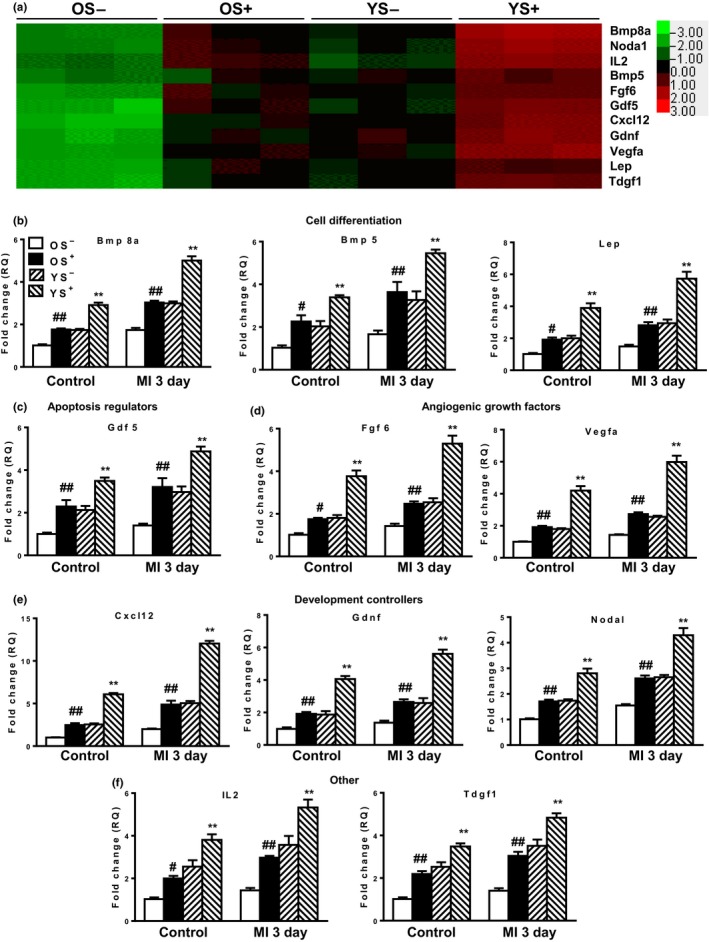
Expression profile of growth factors in cardiac homed donor BM cells. Four months after BM reconstitution, myocardial infarction (MI) was induced and donor BM cells (GFP^+^) were isolated from chimeric hearts at baseline (control without MI) and at 3 days post‐MI. (a) Growth factor qPCR array to profile 84 different growth factors in control groups. Eleven factors were differentially expressed in the homed donor BM cells from the four chimeric groups. The 11 factors were further validated by real‐time qPCR at control as well as at 3 days post‐MI for the4 chimeric groups. These 11 factors were clustered into 5 functional subcategories: (b) cell differentiation regulators (Bmp8a, Bmp5, Lep), (c) apoptosis regulators (Gdf5), (d) angiogenic growth factors (Fgf6, Vegfa), (e) development controllers (Cxcl12, Gdnf, Nodal), and (f) others (IL2, and Tdgf1). *n* = 3/group for the growth factor qPCR array; *n* = 6/group for the other experiments; **p < .01 YS^+^ vs. other group; ^##^ *p* < .01 OS^+^ vs. OS^−^; ^#^ *p* < .05 OS^+^ vs. OS^−^

To identify possible factors responsible for the rejuvenation and regeneration of the aged heart, MI was induced in the four groups of chimeric mice. Donor BM cells (GFP^+^) were isolated from chimeric hearts at baseline and at 3 days post‐MI. And a growth factor qPCR array was carried out to profile 84 different growth factors in the homed donor BM cells from the control groups (BM reconstituted mice without MI, Figure S6), including differentiation, development, and apoptosis regulators (compiled in Figure S7), as well as angiogenic growth regulators and other growth factors (compiled in Figure S8). Eleven factors were identified as significantly differentially expressed by qPCR microarray in the control groups (Figure 5a). These 11 factors were further validated by real‐time qPCR at control as well as at 3 days post‐MI for the four chimeric groups. These 11 factors were clustered into 5 functional subcategories: cell differentiation regulators (bone morphogenetic protein 8a (Bmp8a), bone morphogenetic protein 5 [Bmp5], and leptin [Lep], Figure 5b); apoptosis regulators (growth differentiation factor 5 [Gdf5], Figure 5c); angiogenic growth factors (fibroblast growth factor 6 [Fgf6], vascular endothelial growth factor A [Vegfa], Figure 5d); development controller (chemokine [C‐X‐C motif] ligand 12 [Cxcl12], glial cell line derived neurotrophic factor [Gdnf], nodal homolog [Nodal], Figure 5e); and others (interleukin 2 [IL2] and teratocarcinoma‐derived growth factor 1 [Tdgf1], Figure 5f). All of these regulators were significantly elevated in the donor BM cells derived from OS^+^ and YS^−^ relative to OS^−^ chimeric hearts. However, donor BM cells derived from YS^+^ chimeric hearts had the highest level of expression. MI further amplified the expression of all of these regulators, but the differential expression pattern among the four chimeric groups was similar to that observed at baseline.

**Figure 6:**
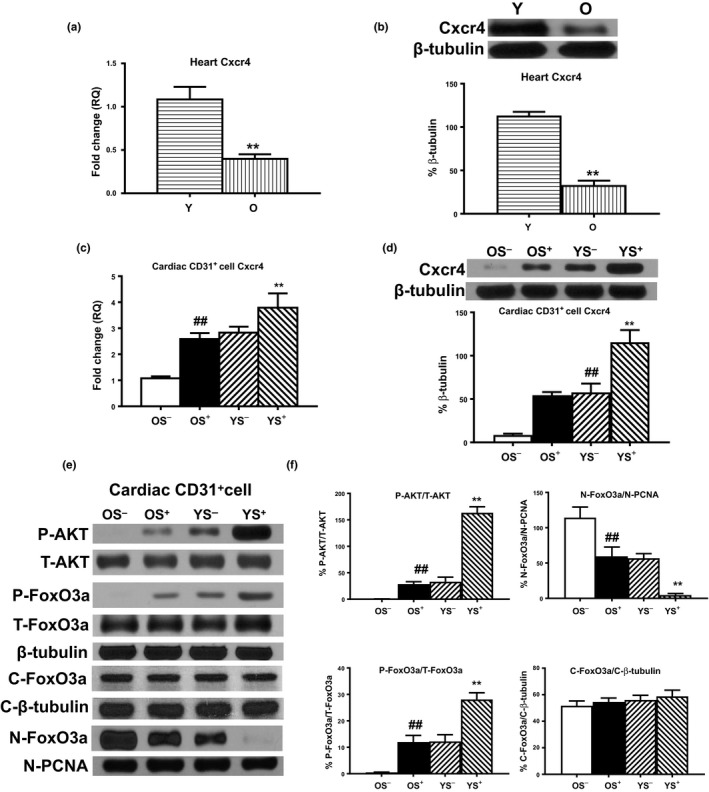
Young BM Sca‐1 cells decreased senescence of aged recipient cardiac endothelial cells through Cxcl12/Cxcr4 pathway. (a) Gene and (b) protein expression of Cxcr4 was compared in the Y and O wild‐type mouse hearts. Four months after BM reconstitution, recipient cardiac endothelial cells were isolated from reconstituted mouse hearts. (c) Gene and (d) protein expression of Cxcr4 and (e) phosphorylated (P−) and total (T−) AKT, P− and T−, as well as cytoplasmic (C−) and nucleus (N−) forkhead box O3a (FoxO3a). (f) Ratios of P‐AKT/T‐AKT, P‐Fox3a/T‐Fox3a, nuclear (N)‐FoxO3a/N‐PCNA, and cytoplasmic (C)‐FoxO3a/C‐β‐tubulin in recipient cardiac endothelial cells from the four chimeric hearts. *n* = 6/group; **p < .01 Y vs. O; YS^+^ vs. other group; ^##^ *p* < .01 OS^+^ vs. OS^−^

Among these 11 regulators, Cxcl12 was identified as the most dramatically increased in the GFP^+^ cells isolated from the YS^+^ chimeric hearts, especially after the induction of MI. Cxcl12 is of interest because it plays an important role in BM stem cell retention, mobilization, homing, and survival (Geng et al., 2015). Cxcl12 exerts its effects by binding the receptors Cxcr7 and Cxcr4, for which it is a unique ligand. To determine whether the effects of BM Sca‐1 cells on recipient endothelial cells are mediated through the Cxcl12‐Cxcr4 axis, we compared the gene and protein expression of Cxcr4 in young and old mouse hearts. Both the gene and protein expression of Cxcr4 was decreased in old mouse hearts (Figure 6a,b). The recipient cardiac endothelial cells from the hearts of the OS^−^ group had the lowest gene and protein expression of Cxcr4 (Figure 6c,d). In response to the increased level of Cxcl12, the gene and protein expression of Cxcr4 was increased in the OS^+^ and YS^−^ groups, with the highest level of expression observed in the YS^+^ group (Figure 6c,d). The protein expression of Cxcr4 downstream mediators, total AKT (T‐AKT) and phosphorylated AKT (P‐AKT), total forkhead box O3a (T‐FoxO3a), and phosphorylated FoxO3a (P‐FoxO3a), was also examined (Figure 6e). Both the ratios of P‐AKT/T‐AKT and P‐FoxO3a/T‐FoxO3a were increased in the recipient cardiac endothelial cells from the OS^+^ and YS^−^ groups relative to the OS^−^ group, with the highest ratios observed in the YS^+^ group (Figure 6f). As a further confirmation, the nuclear protein level of FoxO3a (N‐FoxO3a/N‐PCNA) was decreased in the recipient cardiac endothelial cells from the OS^+^ and YS^−^ groups relative to the OS^−^ group, with the lowest level observed in the YS^+^ group. There were no changes in the cytoplasmic protein level of FoxO3a (C‐FoxO3a/C‐β‐tubulin) among the four chimeric groups, suggesting that young BM Sca‐1^+^ cells increased FoxO3a phosphorylation which resulted in its nuclear exclusion and possibly its eventual ubiquitin proteasome degradation. Taken together, these results suggest that young BM Sca‐1 cells decreased the senescence of aged recipient cardiac endothelial cells through activation of the Cxcl12/Cxcr4 pathway.

To provide direct evidence of the protective role of cardiac BM‐derived Sca‐1^+^ cells, CD31^+^ endothelial cells were isolated from O mouse hearts. Conditioned medium was produced from Y and O BM‐derived Sca‐1^+/−^ cells under hypoxia condition for 24 hr. The expression levels of the senescent markers (p16^INK4a^, p27^Kip1^) were lower and the rejuvenation markers (Bmi1, Cbx8, PNUTS, Sirt1) were higher in BM Sca‐1^+^ cell (or conditioned medium) treated compared to the BM Sca‐1^−^ (or conditioned medium) treated endothelial cells, especially in the YS^+^ group (Figure S9). Since the effects were comparable between direct co‐culture with BM Sca‐1^+/−^ cells or using conditioned medium‐derived from BM Sca‐1^+/−^ cells, subsequent experiments were tested in aged cardiac endothelial cells treated only with Y or O BM Sca‐1^+^ cell‐derived conditioned medium. To prove that Cxcl12 is the key mediator responsible for BM Sca‐1^+^ cell‐elicited cardiac rejuvenation, aged cardiac endothelial cells were treated with Y and O BM Sca‐1^+^‐derived conditioned medium in the presence of a Cxcl12 neutralize antibody. As shown in Figure S10, the protective effects of YS^+^ were almost lost in the presence of a Cxcl12 neutralize antibody with the expression levels of the senescent markers (p16^INK4a^, p27^Kip1^) reversed and the rejuvenation markers (Bmi‐1, CBX8, PNUTS, Sirt1) suppressed in the aged cardiac endothelial cells. Consistent with these findings, the protein expression of p16^INK4a^ and p27^Kip1^ was lowest in the YS^+^ group, was comparably lower in the OS^+^ group, and was the highest in both groups when a Cxcl12 antibody is presented. In contrast, the protein expression of CDK2 and CDK4 in treated aged cardiac endothelial cells showed the opposite pattern (Figure S10B). Telomerase activity and telomerase‐related protein expression were highest in the aged cardiac endothelial cells of the YS^+^ group, comparably higher in the OS^+^ group, and lowest in both groups when a Cxcl12 antibody is presented (Figure S10C,D). On the other hand, to confirm the association of Cxcl12/Cxcr4 is critical for the cardiac rejuvenation effects of BM Sca‐1^+^ cells, aged cardiac endothelial cells were treated with Y or O BM Sca‐1^+^ cell‐derived conditioned medium in the absence or presence of a Cxcr4 blocker. The protein expression of Cxcr4 downstream mediators, total AKT (T‐AKT) and phosphorylated AKT (P‐AKT), total forkhead box O3a (T‐FoxO3a), and phosphorylated FoxO3a (P‐FoxO3a), was examined (Figure S11). Both the ratios of P‐AKT/T‐AKT and P‐FoxO3a/T‐FoxO3a were highest in the aged cardiac endothelial cells of the YS^+^ group, comparably higher in the OS^+^ group, and lowest in both groups when a Cxcr4 blocker is presented. This result clearly provided direct evidence of the protective role of BM‐derived Sca‐1^+^ cells on recipient cardiac endothelial cell senescence and function and identified Cxcl12 as a key modulator of the interaction between homed BM Sca‐1^+^ cells and the Cxcr4 receptor in recipient cardiac endothelial cells which led to the activation of the senescence‐suppressing signal pathway AKT‐FoxO3a‐P27.

### 2.6 Young BM Sca‐1 cells preserved aged mouse cardiac function after MI

We induced MI by LAD ligation in the 4 chimeric groups. Cardiac function was evaluated by echocardiography at 0 (before MI), 7, 14, 21, and 28 days after MI (Figure S12A). After MI, there was a significant decrease in fractional shortening (FS, Figure S12B) and ejection fraction (EF, Figure S12C) and a converse increase in left ventricular internal end‐systolic dimension (LVIDs, Figure S12D) and left ventricular internal end‐diastolic dimension (LVIDd, Figure S12E) in all the groups. However, BM Sca1^+^ cell reconstituted aged mice had better preservation of cardiac function when compared with BM Sca1^−^ cell reconstituted groups with the results showing that both FS and EF were highest in the YS^+^, comparably lower in the OS^+^ and YS^−^, and lowest in the OS^−^ from 14 to 28 days post‐MI. The opposite pattern was observed for LVIDs and LVIDd. Along the same line, the infarct area at 28 days after MI was significantly smaller in BM Sca1^+^ cell reconstituted when compared to BM Sca1^−^ cell reconstituted aged mice, especially in the YS^+^ group (Figure S12F‐G). On the other hand, the scar thickness was significantly higher in the BM Sca1^+^ cell reconstituted aged mice, especially in the YS^+^ group (Figure S12H). These data suggested that cardiac‐resident young BM Sca‐1^+^ cells, through decreasing recipient cardiac endothelial cell senescence and improving cellular survival and function, enabled the global rejuvenation of the aged hearts which also translated to the protection of cardiac function after injury.

## 3 DISCUSSION

Previously, we found that functional recovery was improved in old mice after BM reconstitution with young donor cells (Li et al., 2013) and this minority cardiac cell population elicited its effects through paracrine‐directed improvement in survival, proliferation, and neovascularization. Subsequently, BM Sca‐1 cells were identified as the key BM cell type involved in the rejuvenation of the aged heart (Li et al., 2017). We demonstrated that BM chimerism established in aged mice with a Sca‐1^+^ subset of young cells was associated with better restoration of myocardial progenitors and improved healing of the aged heart by stimulating cell proliferation through activation of the PDGFRβ‐Akt/p27^Kip1^ signaling pathway. Extending from this previous work, in the present study, we showed that 4 months after the transplantation of BM Sca‐1 stem cells into aged recipients, (a) the expression of rejuvenation‐related genes and proteins was increased, (b) the expression of senescence‐related genes and proteins was decreased, and (c) telomerase activity and telomerase‐related protein expression were increased in Sca‐1^+^ chimeric hearts, especially in the young Sca‐1^+^ group. Recipient cardiac endothelial cells were identified as the primary cell type rejuvenated by young BM Sca‐1^+^ cells. The proliferation, migration, and tubular formation abilities of recipient cardiac endothelial cells were preserved by BM Sca‐1^+^ cells*.* Homed donor BM cells secreted more growth factors in aged recipient hearts, especially after the induction of MI. Among the multiple upregulated factors, Cxcl12 was identified as the most dramatically increased factor in the homed donor BM cells isolated from the YS^+^ chimeric hearts, especially after the induction of MI, compared with the other groups. In response to the increased level of Cxcl12, the protein expression of the Cxcr4 receptor and the downstream mediator, Akt, was increased in the recipient cardiac endothelial cells, especially in the young Sca‐1^+^ group. We thus showed that reconstitution of aged BM with young Sca‐1^+^ cells promoted rejuvenation of endothelial cells in the aged heart through activation of the Cxcl12/Cxcr4 pathway.

It has been suggested that chronological age is associated with telomere shortening in cardiac stem cells (CSCs), leading to the inheritance of short telomeres and quick progression to a senescent phenotype in newly formed cardiomyocytes. Senescence of CSCs and myocytes predisposes the development of an aging myopathy. However, in the current study, we found that cardiac endothelial cells were the primary cell type most susceptible to senescence during mouse heart aging and chronological aging coincided mainly with endothelial senescence. We postulated that the status of endothelial cells, which may originate from c‐Kit^+^ cells during development, was the major determinant of cardiac senescence and aging. Indeed, several recent preclinical studies have established endothelial dysfunction as one of the key vascular modifications that occurs during aging resulting in a predisposition for cardiovascular disease (Lakatta & Levy, 2003). Therefore, rejuvenation of aged endothelial cells could be a means by which to counteract cardiac senescence and aging. In fact, we found that BM Sca‐1 cells, through decreasing endothelial senescence and improving endothelial function, effectively decreased global senescence in aged recipient hearts.

CXCL12 and its receptor CXCR4 play a crucial role in the homing of stem and progenitor cells in the BM and control their mobilization into peripheral blood and tissue. Under physiological conditions, a small number of hematopoietic stem and progenitor cells (HSPCs) constantly circulate from the BM to the blood and back through CXCL12 secreted by endothelial cells in the BM triggering the arrest of CXCR4^+^ HSPCs (Mazo, Massberg, & von Andrian, 2011). In conditions of stress or injury, HSPCs lose their anchorage in these niches and are increasingly mobilized into the circulation because of the increased plasma level of CXCL12, which may favor CXCL12‐induced migration of HSPCs into the circulation (Mazo et al., 2011). Numerous studies have revealed that myocardial ischemia significantly upregulates CXCL12 (Hu et al., 2007) which then exerts a protective effect through CXCL12/CXCR4 signaling on resident cardiomyocytes.

Recent studies have found that aging changes the expression of Cxcl12 and Cxcr4 or the response to Cxcl12. Xu et al. (2011) showed that the expression of Cxcl12 was decreased in both the serum and BM of aged ApoE^−^/^−^ mice. Accordingly, Cxcr4 expression in the BM cells of aged ApoE^−^/^−^ mice was also decreased, and BM cell engraftment was impaired which may contribute to the progression of atherosclerosis in ApoE^−^/^−^ mice (Xu et al., 2011). In agreement, Zhang et al. (2011) showed that the expression of Cxcl12 was significantly inhibited in the peripheral blood and burn wounds of old mice. This inhibited expression was associated with impaired perfusion and vascularization of burn wounds with significantly reduced mobilization of BM‐derived angiogenic cells bearing the cell surface molecules Cxcr4 and Sca‐1 in the mouse full‐thickness burn wound model. In our study, we found that in response to the increased level of Cxcl12 from BM Sca‐1^+^ cells, the protein expression of receptor Cxcr4 was increased in recipient cardiac endothelial cells, especially in the young Sca‐1^+^ group. Furthermore, Cxcl12‐Cxcr4 interaction led to activation of AKT and the subsequent phosphorylation and nucleus exclusion of FoxO3a in recipient cardiac endothelial cells.

Activation of AKT induces pro‐survival signaling and inhibits activation of pro‐apoptotic signaling molecules including the FoxO3a transcription factor. Brunet et al. demonstrated that AKT can phosphorylate FoxO3 both in vitro and in vivo on the three predicted sites, T32, S253, and S315, and this phosphorylation resulted in its subsequent cytoplasmic sequestration and/or degradation via the ubiquitin–proteasome pathway (Brunet et al., 1999). As FOXO proteins have been suggested to transcriptionally regulate cell cycle inhibitory genes (e.g., p27^kip1^), the inhibition of these transcription factors and the consequent inhibition of cell cycle inhibitors would contribute to extensive cellular proliferation. Most previous studies have focused on the role of AKT‐mediated FoxO3a inhibition of tumor development (Tzivion, Dobson, & Ramakrishnan, 2011). To our knowledge, our study is the first to report that homed BM Sca‐1^+^ cells phosphorylate AKT in aged recipient cardiac endothelial cells through Cxcl12‐Cxcr4 interactions. The subsequent phosphorylation and cytoplasmic accumulation of FoxO3a led to downregulation of the cell cycle inhibitor p27^Kip1^. The inhibition of this transcription factor and the consequent inhibition of the cell cycle inhibitor contributed to decreased recipient cardiac endothelial cell senescence and improved cellular survival and function. Such effects promote the global rejuvenation of aged Sca‐1^+^ recipient hearts.

Recently, we evaluated the importance of the function of cardiac‐resident BM‐derived progenitors, especially the Sca‐1^+^ cells on cardiac recovery after injury in aged animals (Li et al., 2017). At homeostasis, these BM Sca1^+^ cells maintained the monocyte/progenitor cell pool in aged mouse heart. After MI, these homed BM Sca‐1^+^ cells stimulated proliferation of donor and host progenitor cells in the aged heart which was associated with better restoration of cardiac function. However, these cardiac‐resident BM‐derived cells did not participate directly in angiogenesis or cardiomyogenesis through differentiation. We believed that the improvement in cardiac function resulted from stimulation of other cells within their sphere of influence through paracrine factors, thereby amplifying their beneficial effects beyond their number and location. Indeed, we found a variety of cardioprotective factors (Cxcl12, Vegfa, Fgf 6, Gdf5) were significantly higher in the Sca‐1^+^ subset. We showed that a significant increase in rejuvenating and angiogenic factors combine through downstream AKT activity and the related pro‐survival and pro‐vascular factors contributed to the protection of the heart. Although the mechanisms and the exact mediators underlying BM Sca‐1^+^ cell‐mediated cardiac rejuvenation of the aged heart remain elusive, we believe this minority cardiac cell population elicited its effects through paracrine‐directed improvement in rejuvenation, survival, proliferation, and neovascularization.

## 4 CONCLUSIONS

We demonstrated the effects of BM Sca‐1^+^ cells on rejuvenating the aged heart using in vivo BM reconstitution and in vitro cell functional assays. We identified Cxcl12 as a key modulator of the interaction between homed BM Sca‐1^+^ cells and the Cxcr4 receptor in recipient cardiac endothelial cells which led to activation of the senescence‐suppressing signal pathway AKT‐FoxO3a‐P27. Activation of this pathway contributed to decreased recipient cardiac endothelial cell senescence and improved cellular survival and function, enabling the global rejuvenation of the aged heart following BM reconstitution with Sca‐1^+^ cells.

## 5 EXPERIMENTAL PROCEDURES

### 5.1 Animal procedures

The Animal Care Committee of the University Health Network approved all experimental procedures which were carried out according to the Guide for the Care and Use of Laboratory Animals (NIH, revised 2011). BM cells from young (Y, 2–3 months) or old (O, 18–19 months) GFP^+^ transgenic mice (C57BL/6‐Tg‐GFP mice, The Jackson Laboratory) were flushed from the tibias and femurs, and mononuclear cells were separated by density gradient centrifugation and then separated into Sca‐1^+^‐ and Sca‐1^−^‐labeled fractions by immunomagnetic‐activated cell sorting (Stem Cell Technology, Cat#: 18756). Female C57BL/6 mice aged 18–19 months (old [O] recipients) were lethally irradiated (9.5 Gy) and immediately received an infusion (through the tail vein) of fresh Sca‐1^+^ or Sca‐1^−^ BM cells (2 × 10^6^) from young or old donor mice, generating Y(Sca1^+^)‐O, Y(Sca1^−^)‐O, O(Sca1^+^)‐O, and O(Sca1^−^)‐O chimeras as described previously (Li et al., 2017). All aged chimeric mice were housed for 4 months to allow young/old stem cells to repopulate the BM and home to the heart prior to further experimentation. Thus, coronary occlusion was performed on chimeric mice 4 months after BM reconstitution and heart tissues were used for various analyses at the gene, protein, and cellular function levels.

For myocardial infarction, coronary occlusion was performed in Y(Sca‐1^+^)‐O, Y(Sca‐1^−^)‐O, O(Sca1^+^)‐O, and O(Sca1^−^)‐O chimeric mice 4 months after BM reconstitution, as previously reported (Li et al., 2013). In brief, mice were intubated and ventilated with 2% isoflurane and given buprenorphine (0.05 mg/kg, S.C.) for analgesia. Through a thoracotomy, the pericardium was dissected and the left anterior descending (LAD) coronary artery was ligated.

More detailed experimental procedures can be found in the Supporting Information file.

### 5.2 Statistics

All values are expressed as mean ± *SD*. Analyses were performed using GraphPad InStat software . Student's *t* test was used for two‐group comparisons. Comparisons of parameters among three or more groups were analyzed using one‐way analysis of variance (ANOVA) followed by Tukey or two‐way ANOVA with repeated measures over time followed by Bonferroni post hoc tests for multiple comparisons. Differences were considered statistically significant at *p* < .05.

## CONFLICT OF INTEREST

The authors have no conflicts of interest to declare.

## AUTHOR CONTRIBUTIONS

JL, JD, Z‐BS, H‐FS, W‐JY, SE, and JW conducted the experiments described in the paper; JL wrote the manuscript; S‐HL contributed to the design of the study, analysis, and interpretation of the data, and revised the manuscript; F‐JA and C‐YZ contributed to the paper revision; RDW, S‐ML, and R‐KL designed the study and wrote the manuscript.
