# Supplementary material for: Long‐term repopulation of aged bone marrow stem cells using young Sca‐1 cells promotes aged heart rejuvenation
Source: Aging Cell. 2019 Aug 5;18(6):e13026. doi: 10.1111/acel.13026 (PMC6826122; doi:10.1111/acel.13026)
Supplement: Supplementary file 1 [file ACEL-18-e13026-s001.docx]

**SUPPORTING INFORMATION**

for

**Long-Term Repopulation of Aged Bone Marrow Stem Cells Using Young Sca-1 Cells Promotes Aged Heart Rejuvenation**

Jiao Li^1,2^, Shu-Hong Li^2^, Jun Dong ^1,2^, Faisal J. Alibhai^2^, Chongyu Zhang^1,2^, Zheng-Bo Shao^2^, Hui-Fang Song^2^, Sheng He^2^, Wen-Juan Yin^2^, Jun Wu^2^, Richard D. Weisel^2,3^, Shi-Ming Liu^1#^, Ren-Ke Li^2,3#^

^1^Guangzhou Institute of Cardiovascular Disease, Guangdong Key Laboratory of Vascular Diseases, State Key Laboratory of Respiratory Disease, the Second Affiliated Hospital, Guangzhou Medical University, Guangzhou, China

^2^Toronto General Hospital Research Institute, Division of Cardiovascular Surgery, University Health Network, Toronto, Canada

^3^Division of Cardiac Surgery, Department of Surgery, University of Toronto, Toronto, Canada

**^#^Addresses for correspondence:**

Ren-Ke Li, MD, PhD

PMCRT, Room 3-702

101 College Street, Toronto, Ontario, Canada M5G 1L7

Tel: 1-416-581-7492; Fax: 1-416-581-7493; [renkeli@uhnresearch.ca](mailto:renkeli@uhnresearch.ca)

Shi-Ming Liu, MD

Department of Cardiology, Second Affiliated Hospital of Guangzhou Medical University,

Guangzhou 510260, China

Tel: 86-020-34153522; Fax: 86-20-3415-3709; [gzliushiming@126.com](mailto:gzliushiming@126.com)

**Experimental Procedures**

***Isolation and culture of cardiac cells***

Young (Y, 2-3 mos) and old (O, 22-23 mos) wild type mice (C57BL/6, The Jackson Laboratory) were used to isolate cardiomyocytes, endothelial cells and aortic smooth muscle cells (SMC). The method previously described by Zhou et al. was adopted with modifications for cardiomyocyte isolation ([Zhou et al., 2000](#_ENREF_4)). In brief, mice were intubated and ventilated with 2% isoflurane. Through a thoracotomy, the heart was quickly removed from the chest and retrogradely aortic perfused at a constant pressure (100 cm H_2_O) at 37°C for ~3 min with a Ca^2+^-free bicarbonate-based buffer as described by Zhou et al.([Zhou et al., 2000](#_ENREF_4))(1). The enzymatic digestion was initiated by adding collagenase type II (1 mg/ml; Worthington Biochemical Corp., Cat#:LS004176), to the perfusion solution. When the heart became swollen and hard after ~3 min of digestion, 50 mM Ca^2+^ was added to the enzyme solution. About 7 min later, the left ventricle was quickly removed, cut into several chunks, and further digested in the same enzyme solution. The supernatant containing the dispersed myocytes was subjected to a 3-step Ca^2+^ restoration and contained 125, 250, and 500 mM Ca^2+^, respectively. Freshly isolated cardiac myocytes were plated on laminin-coated dishes with minimal essential medium (MEM; Sigma M1018) containing 1.2 mM Ca^2+^, 2.5% fetal bovine serum (FBS; GIBCO), and 1% PS (pH 7.35–7.45). After 1 h of culture in a 5% CO_2_ incubator at 37^º^C, the medium was changed to FBS-free MEM and the cells were used within 6-8 hours after isolation. For cardiac endothelial isolation, after being well perfused as described above, hearts were quickly removed, cut into small chunks, and further digested in 1 mg/ml of collagenase type II for 30 to 45 min. The supernatant containing the dispersed cells was filtered into a sterilized tube and gently centrifuged at 1000 rpm for 5 min. The cell pellet was then promptly resuspended in medium buffer (PBS+2% FBS+1 mM EDTA) and subjected to subsequent immunomagnetic activated cell sorting using PE conjugated anti-mouse CD31 antibody (BD Bioscience, Cat#:561073) and the PE isolation kit (Stem Cell Technology, Cat#:17656). The isolated cells were cultured in endothelial growth medium (Lonza, EGM-2, Cat#: CC3162).

Cardiac fibroblasts were isolated as previously described ([Wang et al., 2014](#_ENREF_3)). Briefly, mouse hearts were digested with 0.1% collagenase type II (Worthington) for 30 min at 37°C. The isolated cells were resuspended in Iscove’s Modified Dulbecco’s Medium (IMDM; Gibco), with 10% FBS, 100 U/mL penicillin G, and 100 μg/mL streptomycin. After 3–5 days of culture, the non-adherent cells were washed off. Adherent cardiac fibroblasts were used for the experiments.

Mouse aortic SMCs were prepared as previously described by Smith and Brock with minor modifications ([Smith & Brock, 1983](#_ENREF_2)). In brief, the transverse and descending aorta were removed and placed in isolation medium consisting of DMEM containing 25 mM HEPES (pH 7.4), 100 pg/L gentamicin, 2.5 pg/mL amphotericin B, and 1 mg/mL bovine serum albumin. Aortic tissue was cleaned of fat and connective tissue and incubated for 30 min at 37°C in isolation medium supplemented with 200 U/mL collagenase type III (Sigma), 0.1 mg/mL elastase (132 U/mg; Sigma), and 0.5 mg/mL soybean trypsin inhibitor (Sigma). After a second digestion of 45 min, the medial tissue was thoroughly washed, minced finely with scissors, and placed in fresh digestion medium. After 1 h, suspended cells were collected. The remaining tissue was transferred to fresh digestion medium and incubated for 1 h at 37°C. Suspended cells were collected by centrifugation at 200 × g for 3 min and plated in DMEM containing 10% FBS, 50 ng/mL platelet-derived growth factor (PDGF; Sigma), 50 pg/L gentamicin, and 2.5 pg/mL amphotericin B. The medium was changed once weekly.

To isolate donor and recipient-derived cells from chimeric hearts at 4 months post-BM reconstitution, the hearts from all four chimeric groups were immediately isolated and processed as described above. Flow cytometry based cell sorting was used to collect the GFP^+^ donor and GFP^-^ recipient cell fractions. Cardiac endothelial cells were isolated from the GFP^-^ fraction by magnetic bead sorting and cultured in endothelial growth medium as described above.

***Real-time reverse transcription-polymerase chain reaction***

The expression of senescence-related genes (p16^INK4a^, p19^ARF^, and p27^Kip1^) and rejuvenation-related genes (Bmi1 polycomb ring finger oncogene [Bmi1], chromobox 8 [Cbx8], protein phosphatase 1, regulatory subunit 10 [PNUTS], sirtuin 1 [Sirt1], sirtuin 2 [Sirt2], and sirtuin 6 [Sirt6]) was evaluated using real-time reverse transcription-polymerase chain reaction. GAPDH was used as a housekeeping gene. In brief, total RNA was isolated with TRIzol reagent (Invitrogen, Grand Island, NY) and cDNA was synthesized using Moloney murine leukemia virus reverse transcriptase and random primers. Real-time polymerase chain reaction was conducted using SensiFAST SYBR Green PCR Master Mix (Bioline USA Inc., Taunton, MA) with the following parameters: 95^o^ 2 min; [95^o^ 5 s; 60^o^ 30 s for 40 cycles]. The oligonucleotide primer sequences are shown in Supplementary Table 1.

***Real-time reverse transcription-polymerase chain reaction array***

Genomic DNA removal, cDNA synthesis, pre-amplification and qRT-PCR were performed using RT2 Profiler PCR Arrays (Qiagen, Cat#: 330522) according to the manufacturer’s instructions. In brief, 300 ng RNA was treated with DNA elimination mix and cDNA was subsequently synthesized. The intended PCR products were pre-amplified, followed by Side Reaction Reducer and heat inactivation. A SYBR Green-based qRT-PCR reaction was performed in duplicate on an Eppendorf Realplex^2^ system. A custom combination of primer sets was used to profile 84 different growth factors. Four reference genes were used for normalization. Averaged duplicate measurements were scaled by standard deviation. Negative genomic DNA contamination and positive reverse transcriptase and PCR controls were included for each sample.

***Immunofluorescent staining***

Hearts were fixed in 2% paraformaldehyde (PFA) for 24 h after being well perfused with PFA and were then stored in 0.5M sucrose at 4^º^C overnight. Hearts were then embedded with OCT, and 5-µm-thick frozen sections were prepared. Slides were incubated with one of the following primary antibodies: Alexa488 conjugated anti-GFP (Invitrogen, Cat#: A21311, 1:400) or Goat anti-GFP (Abcam, Cat#: ab6673, 1:400), anti-VWF (Santa Cruz, Cat#: 14014, 1:100), anti-P16 (Abcam, Cat#: ab54210, 1:100), anti-α-smooth muscle actin (SMA, Sigma, Cat#: A-2547, 1:400), anti-sarcomeric α-actinin (SARC, Abcam, Cat#: ab9465, 1:100), anti-DDR2 (Santa Cruz, Cat#: Sc-7555, 1:50) at room temperature for 2 h. Incubation with respective Alexa 488 or 568 or 647 conjugated secondary antibodies (Invitrogen, all 1:400) was carried out at room temperature with light protection for 1 h. The nuclei were identified with DAPI. The number of positive cells in 6 randomly selected high-power fields per section was determined and quantified as a percentage of total cells (DAPI^+^) in the corresponding high-power fields, then averaged for 6 sections (6 mouse hearts) with a Nikon fluorescent microscope. An Olympus Fluoview 2000 laser scanning confocal microscope was used to confirm the co-localization of fluorescent signals.

***Western blotting***

For Western blotting, 50 μg of lysate was fractionated through a 4% stacking and 10% running SDS-PAGE gel, and the fractionated proteins were transferred to a PVDF membrane. Blots were blocked for 1 h at room temperature with blocking buffer. The antibodies [P16 (Abcam, Cat#: ab54210), P27 (Cell Signalling, Cat#: 9567S), CDK2 (Abcam, Cat#: ab6326), CDK4 (Cell Signalling, Cat#: NB200-169), DNA-PKc (Abcam, Cat#: ab32566), TRF-2 (Santa Cruz, Cat#: sc-9143), Akt (Cell Signaling, Cat#: 9272), Akt Ser 473 (Cell Signaling, Cat#: 9271S), FoxO3a (Abcam, Cat#: ab23683), FoxO3a phospho S253 (Abcam, Cat#: ab31109) all 1:1000] reacted with the blots overnight at 4°C. After washing (3 x 5 min in 1xTBS-0.1% Tween 20), the blots were incubated with horseradish peroxidase-conjugated secondary antibody at 1:2000 dilution for 1 h at room temperature. Visualization was performed with enhanced chemiluminescence. For quantification, densitometry of the target bands was divided by the corresponding densitometry of the GAPDH or β-tubulin band using AlphaImager 2200 software. Cytoplasmic and nuclear extracts were obtained using the NE-PER Nuclear and Cytoplasmic Extraction Kit (Pierce Biotechnology, Rockford, IL, USA) according to the manufacturer’s instructions. In brief, the cell pellet was re-suspended in cytoplasmic extraction reagent (CER) I, vortexed and incubated on ice for 10 min. Then CER II was added, and the extracts were further incubated on ice. Thereafter, the extracts were vortexed again and centrifuged, and the supernatant (cytosolic extract) was transferred to a pre-chilled tube. The nuclear pellet was re-suspended in nuclear extraction reagent.

***Telomerase activity assay***

Telomerase activity was measured by a telomeric repeat amplification protocol (TRAP) assay with a telomerase PCR ELISA kit (TeloTAGGG Telomerase PCR ELISA, Roche, Cat#:11854666910) according to a published protocol ([Du, Li, Lin, & Wu, 2004](#_ENREF_1)). Approximately 2 × 10^5^ cells or 500 µm thick frozen tissue samples were harvested for each reaction and centrifuged at 3000 g for 10 min at 4°C. The absorbance for the final product was acquired by measuring absorbance at 450 nm.

***Co-culture studies***

Conditioned medium was produced from BM Sca1^+^ or Sca1^-^ cells (1x10^5^/cm^2^) in serum-free DMEM medium under hypoxia condition for 24 hours. CD31^+^ endothelial cells were isolated from O mouse hearts, and co-cultured with Y Sca-1^+^ (YS^+^), Y Sca-1^-^ (YS^-^), O Sca-1^+^ (OS^+^), and O Sca-1^-^(OS^-^) BM cells or with the BM cell conditioned medium [respectively](javascript:;) under hypoxia for 72 h. For inhibition studies, either anti-Cxcl12 neutralizing antibody (110 μg/mL, R&D systems, Cat#: MAB310) or Cxcr4 blocker (AMD 3100, 10 μM, Sigma, Cat#: A5602) was added to the cell culture medium.

***Cardiac Endothelial Cell proliferation assay***

Cardiac endothelial from the 4 chimeric groups were isolated and cultured as described above. For BrdU (5-bromo-2'-deoxyuridine, Sigma, Cat#: B5002) labeling, the cells were seeded into 24-well plates (4x10^3^/well) with endothelial basal medium (EBM) overnight and then supplemented with BrdU (10 µM/mL). After BrdU pulse chasing for 48 h, the cells were fixed for immunofluorescent staining with BrdU (Abcam, Cat#: ab6326).

For BrdU immunostaining, the fixed cells were first treated with 2N HCl for 30 min (10 min at RT, 20 min at 37ºC) to expose the antigens and then permeabilized with 0.5% Triton X-100. Sections were incubated with rat anti-BrdU (1:80) for 2 h in a humidified atmosphere. After incubation with the primary antibody, the slides were washed three times in PBS and incubated with an Alexa 568-conjugated goat anti-rat secondary antibody (Invitrogen) for 1 h at room temperature.

***Cell migration assay***

The transwell system was used to investigate cardiac endothelial cell migratory response to vascular endothelial growth factor (Vegf). After 4 days in culture, cardiac endothelial cells from the 4 chimeric groups (2x10^5^/cm^2^) were detached using trypsin/EDTA (GIBCO), harvested by centrifugation, resuspended in 200 μl EBM and plated in the transwell cell culture inserts (8-μm diameter pores). The inserts were placed in a 24-well culture dish containing EBM with 10% FBS and mouse recombinant Vegf (50 ng/ml). After 24 h incubation at 37°C, cells on the upper layer of the insert membrane were completely removed by a cotton swab. Cardiac endothelial cell that migrated to the other side of the membrane were fixed and stained with 0.5% crystal violet.

***In vitro tubule formation assay***

Growth factor reduced matrigel (Becton Dickenson, Cat#: 354230) was thawed and placed in 24-well culture plates at 37^o^C for 30 minutes to allow solidification. Cardiac endothelial from the 4 chimeric groups (5 x 10^4^) suspended with EBM were dispensed on the solidified gel and incubated at 37°C for 24 h. Pictures were taken using a Nikon microscope and the numbers of tubes counted to measure tube-forming ability. Cardiac endothelial cells were cultured for 6 days prior to use in the matrigel assay.

***SA-β-gal staining***

Cells grown in 6-well plates at a density of 3.5×10^3^ cells/cm^2^ were washed with PBS and then stained with the senescence β-galactosidase staining kit (Cell Signaling, Cat#: 9860) according to the manufacturer’s instruction. Briefly, the cells were fixed with the fixing solution for 15 min, then washed with PBS and incubated with the SA- β -gal staining solution (SA- β -gal staining solution A 10 μl, SA- β -gal staining solution B 10 μl, SA- β -gal staining solution C 930 μl and X-gal 50 μl) overnight at 37 °C. Pictures were taken using a Nikon microscope.

**Supplementary Figures and Figure Legends**

**
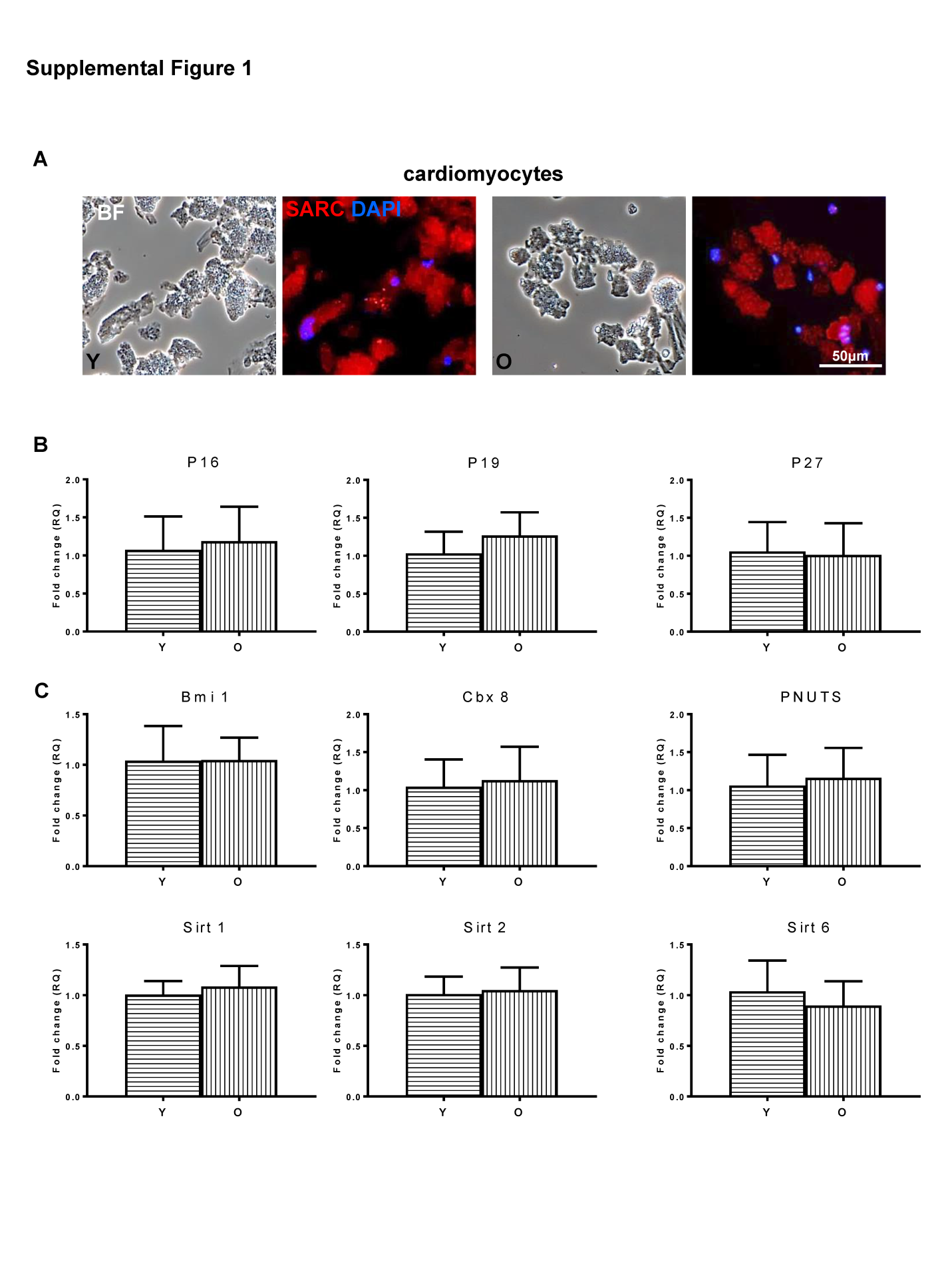
**

**Supplementary Figure 1. Senescent phenotype in cardiomyocytes**

**(A)** Representative bright field (BF) and immunofluorescent (sarcomeric α-actinin, SARC) images of cardiomyocytes isolated from young (Y, 2-3 mos) and old (O, 22-23 mos) wild type (C57BL/6) mouse hearts. **(B)** senescence-related (p16^INK4a^, p19^ARF^, and p27^Kip1^) and **(C)** rejuvenation-related (Bmi1, Cbx8, PNUTS, Sirt1, Sirt2, and Sirt6) genes was not significantly different between Y and O mice when detected by RT-qPCR. n= 6/group; mean±SD.

**
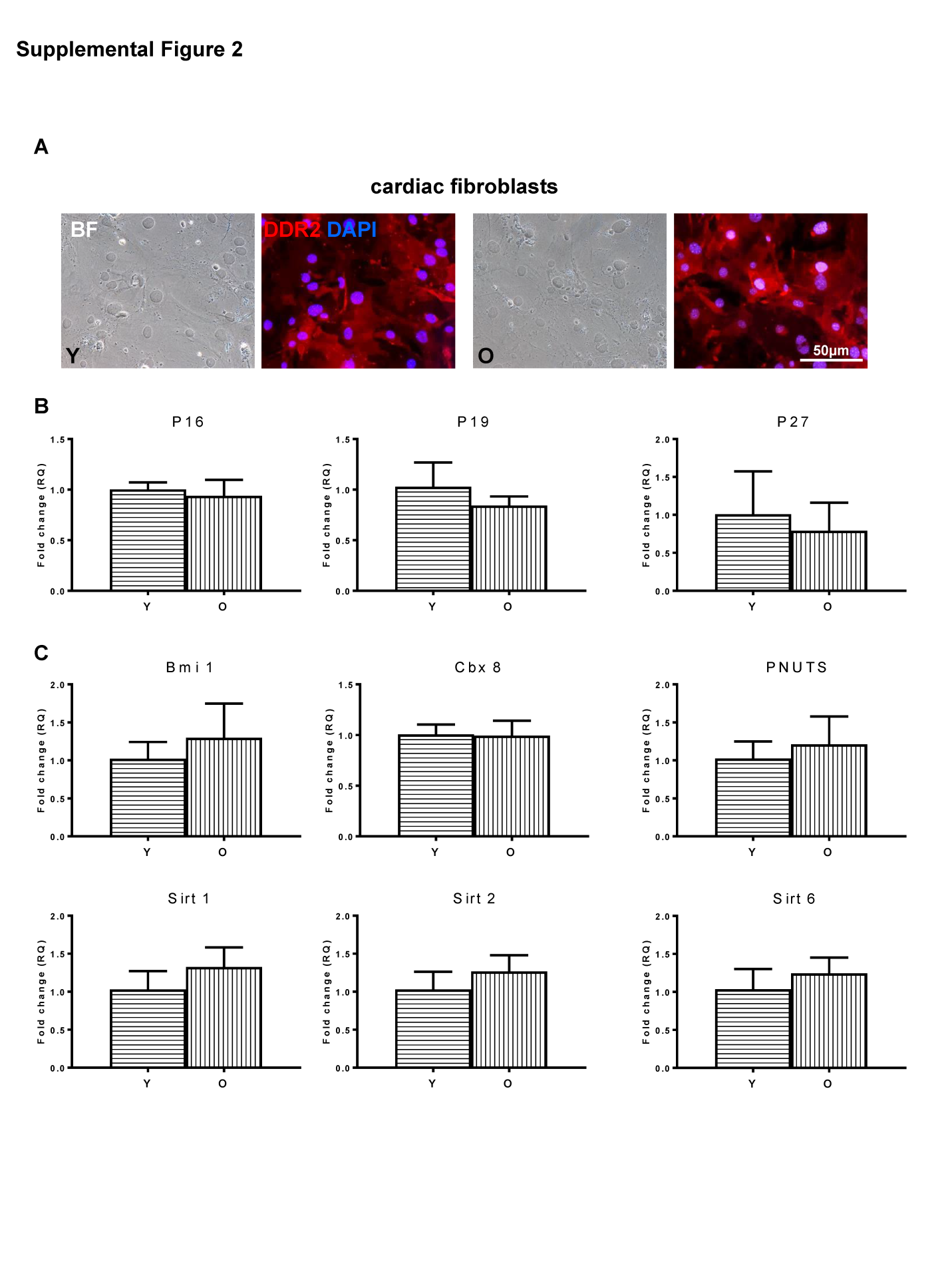
**

**Supplementary Figure 2. Senescent phenotype in fibroblasts**

**(A)** Representative bright field (BF) and immunofluorescent (discoidin domain receptor 2, DDR2) images of fibroblasts isolated from young (Y, 2-3 mos) and old (O, 22-23 mos) wild type (C57BL/6) mouse hearts. **(B)** senescence-related (p16^INK4a^, p19^ARF^, and p27^Kip1^) and **(C)** rejuvenation-related (Bmi1, Cbx8, PNUTS, Sirt1, Sirt2, and Sirt6) gene expression was not found to be significantly different between the Y and O mice when detected by RT-qPCR. n= 6/group; mean±SD.

**
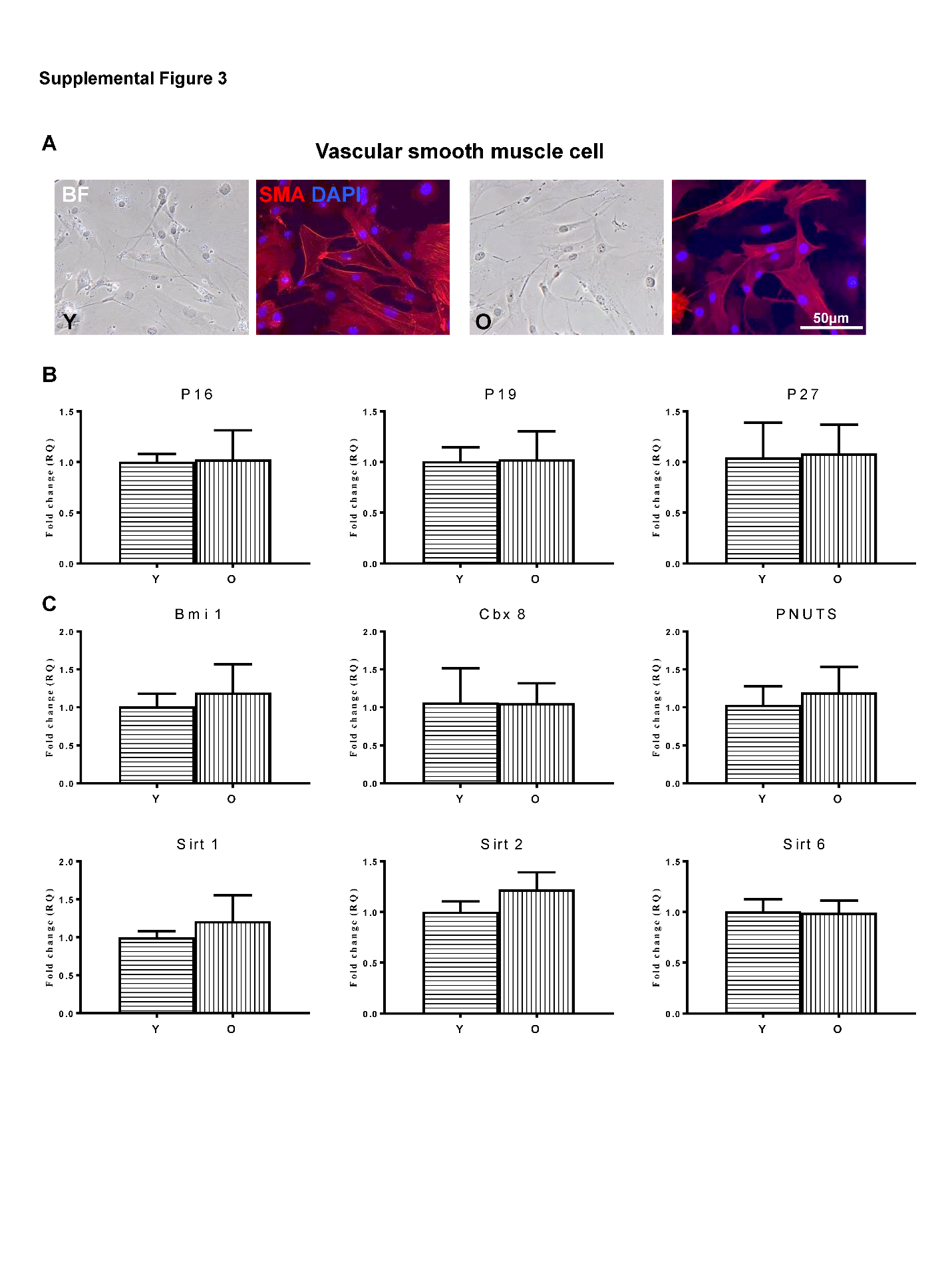
**

**Supplementary Figure 3. Senescent phenotype in vascular smooth muscle cells**

**(A)** Representative bright field (BF) and immunofluorescent (smooth muscle actin, SMA) images of vascular smooth muscle cells (VSMC) isolated from young (Y, 2-3 mos) and old (O, 22-23 mos) wild type (C57BL/6) mouse aorta. **(B)** senescence-related (p16^INK4a^, p19^ARF^, and p27^Kip1^) and **(C)** rejuvenation-related (Bmi1, Cbx8, PNUTS, Sirt1, Sirt2, and Sirt6) gene expression was not found to be significantly different between the Y and O mice when detected by RT-qPCR. n= 6/group; mean±SD.





**Supplementary Figure 4. Number of GFP^+^ cells in the chimeric mouse bone marrow, blood and hearts.**

BM Sca-1^+^ or Sca-1^-^ cells (2X10^6^) from young (Y, 2-3 mos) or old (O, 18-19 mos) GFP transgenic mice were transplanted into lethally-irradiated (9.5 Gy) O mice to generate 4 groups of chimeras: Y Sca-1^+^ (YS^+^), Y Sca-1^-^ (YS^-^), O Sca-1^+^ (OS^+^), and O Sca-1^-^ (OS^-^), respectively. Four months later, GFP^+^ cells in the BM **(A),** blood **(B)** and heart **(C)** of the reconstituted mice were quantified by flow cytometry. n= 6/group; **P*<0.05 YS^+^ *vs* YS^-^, ^#^*P*<0.05 OS^+^ *vs* OS^-^.

**
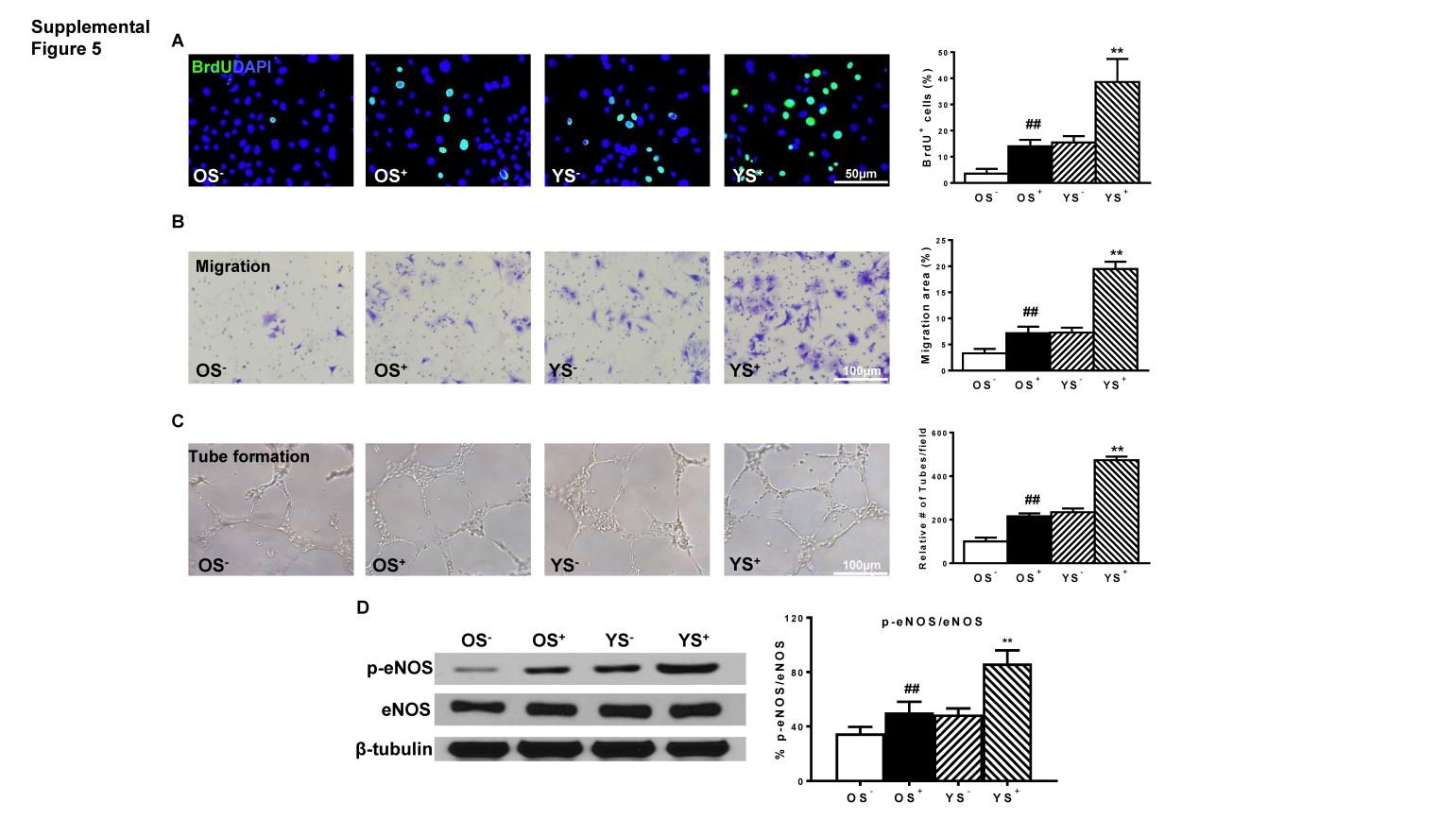
**

**Supplementary Figure 5. Young BM Sca-1 cells preserved recipient cardiac endothelial cell function after BM reconstitution.**

Four months after BM reconstitution, recipient cardiac endothelial cells were isolated from reconstituted mouse hearts. **(A)** BrdU (5-bromo-2'-deoxyuridine) labelling of the proliferative recipient cardiac endothelial cells. **(B)** The migratory ability, **(C)** tubular structure, and **(D)** ratio of phospho-eNOS/total eNOS protein were compared in the recipient cardiac endothelial cells from the 4 chimeric hearts. n= 6/group; **P<0.01 YS^+^ *vs* other group; ^##^P<0.01 OS^+^ *vs* OS^-^.

**
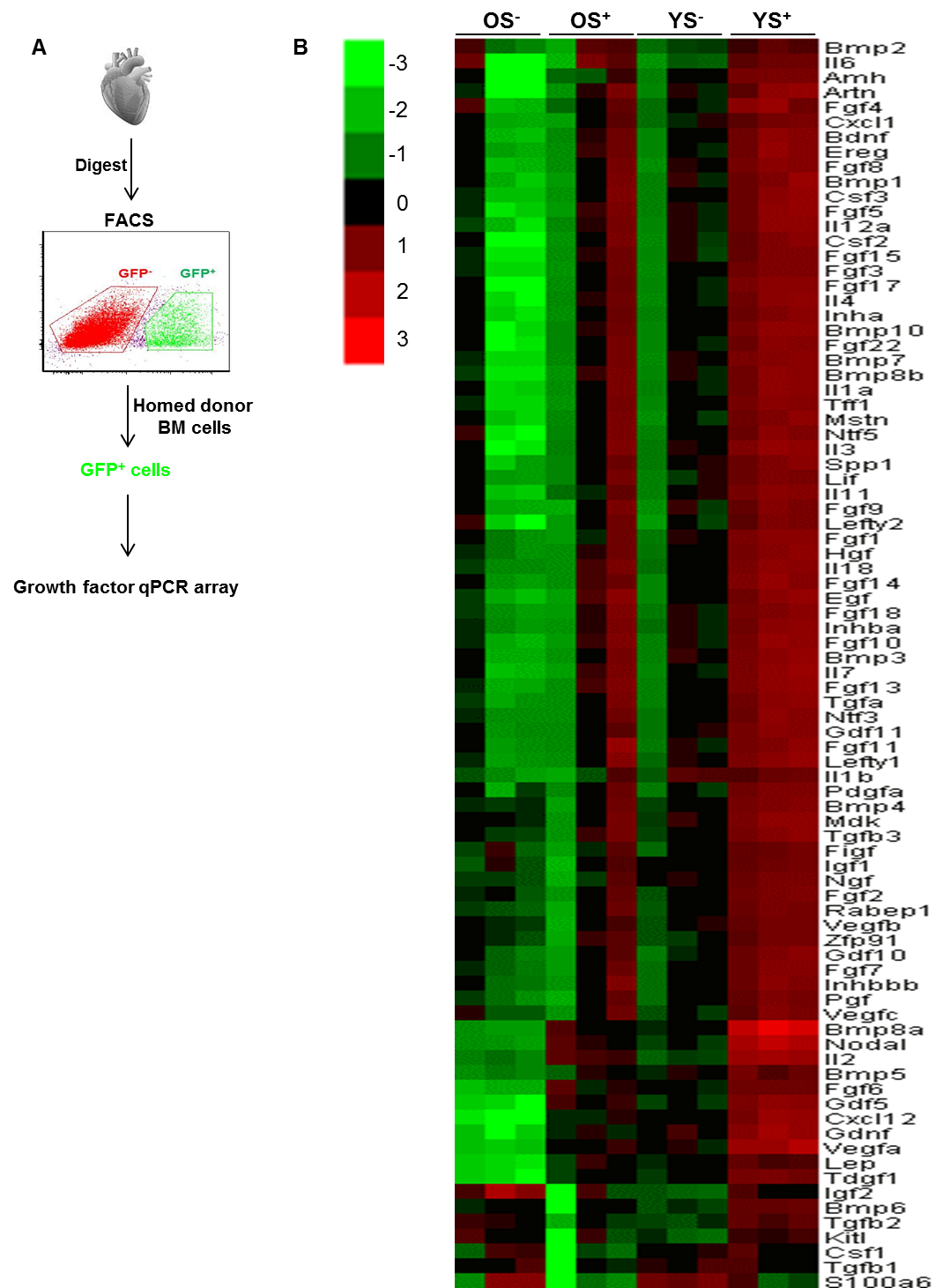
**

**Supplementary Figure 6. Expression profile of different growth factors in cardiac homed donor BM cells**

Four months after BM repopulation, donor BM cells (GFP^+^) were isolated from chimeric hearts using fluorescence-activated cell sorting (FACS) and a growth factor qPCR array was carried out to profile 84 different growth factors. **(B)** Heatmap depicting qPCR microarray gene expression profiles of the four chimeric groups. n= 3/group. OS^-^ indicates O(Sca1^-^)-O, OS^+^ indicates O(Sca1^+^)-O, YS^-^ indicates Y(Sca1^-^)-O, and YS^+^ indicates Y(Sca1^+^)-O chimeras.

**
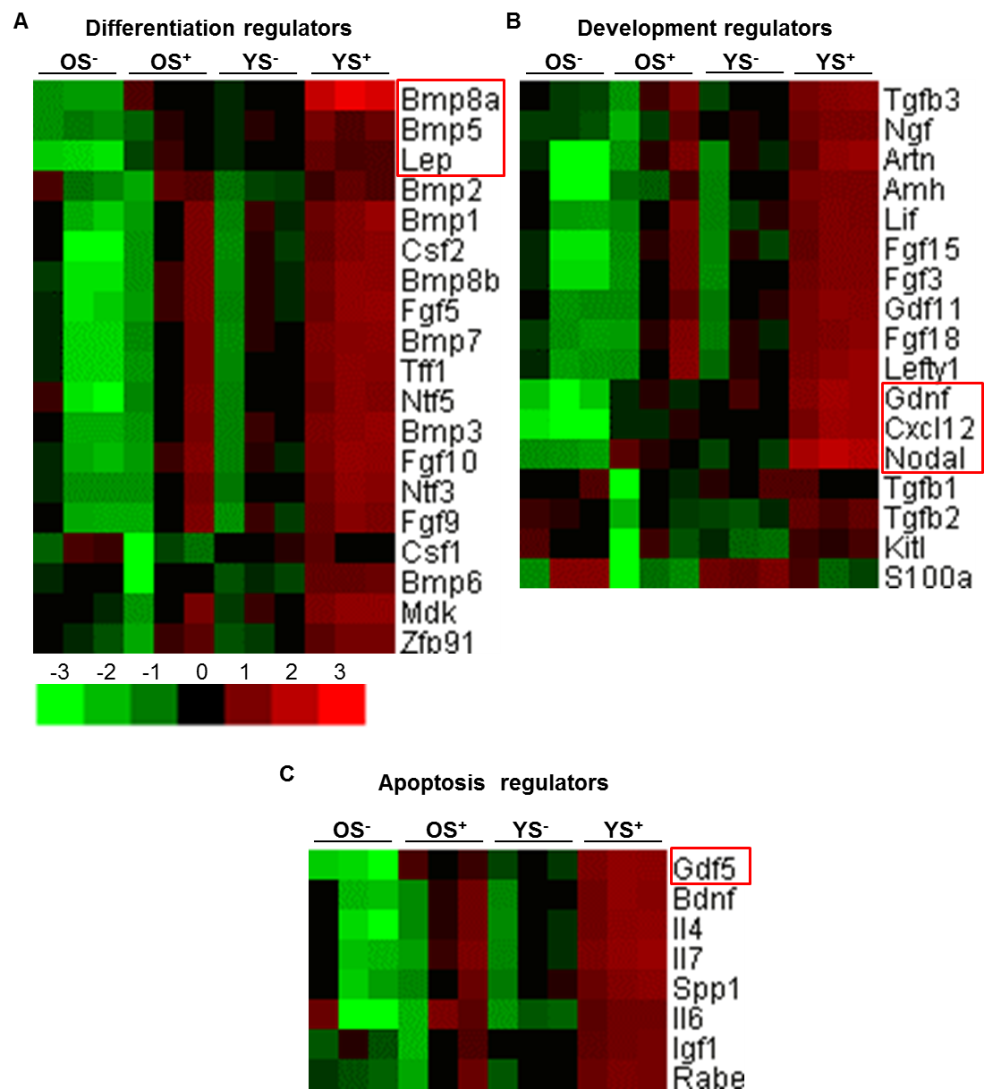
**

**Supplementary Figure 7. Expression profile of cell differentiation, development and apoptosis regulators in cardiac homed donor BM cells**

Homed donor BM cells (GFP^+^) were isolated from chimeric hearts by fluorescence-activated cell sorting (FACS). Heatmap depicting qPRC microarray gene expression profiles of homed donor BM cells (GFP^+^) in the 4 chimeric groups for **(A)** differentiation, **(B)** development and **(C)** apoptosis regulators. n= 3/group.

**
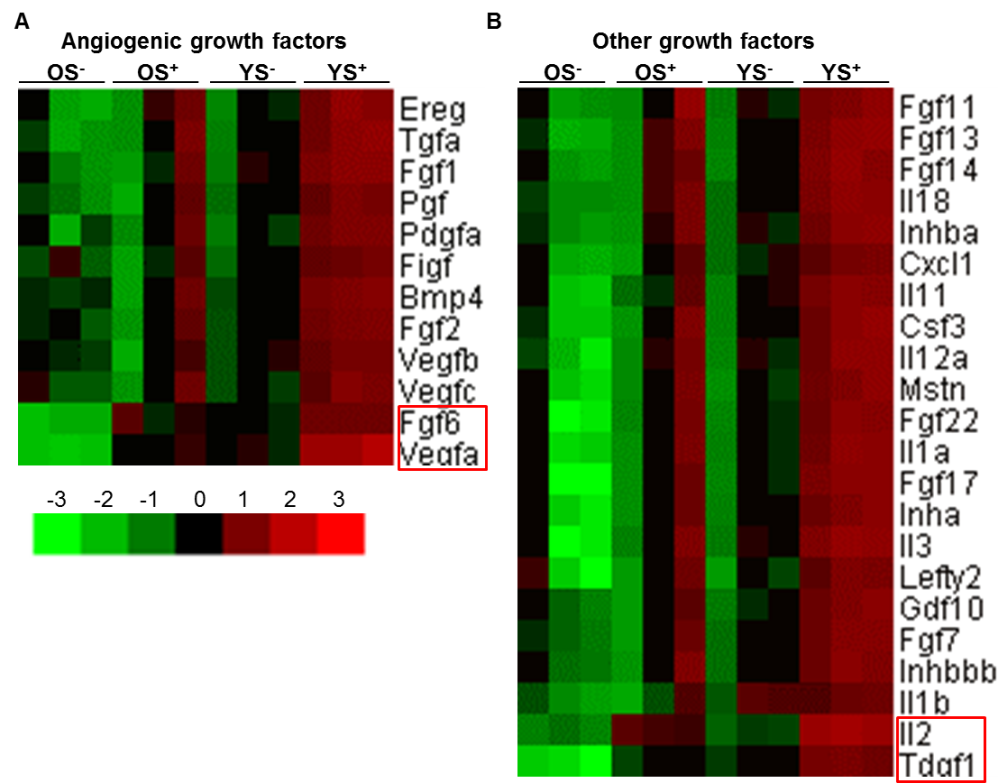
**

**Supplementary Figure 8. Expression profile of angiogenic growth regulators and other growth factors in cardiac homed donor BM cells**

Heatmap depicting qPRC microarray gene expression profiles of homed donor BM cells (GFP^+^) in the four chimeric groups for **(A)** angiogenic growth regulators and **(B)** other growth factors. n= 3/group.

**
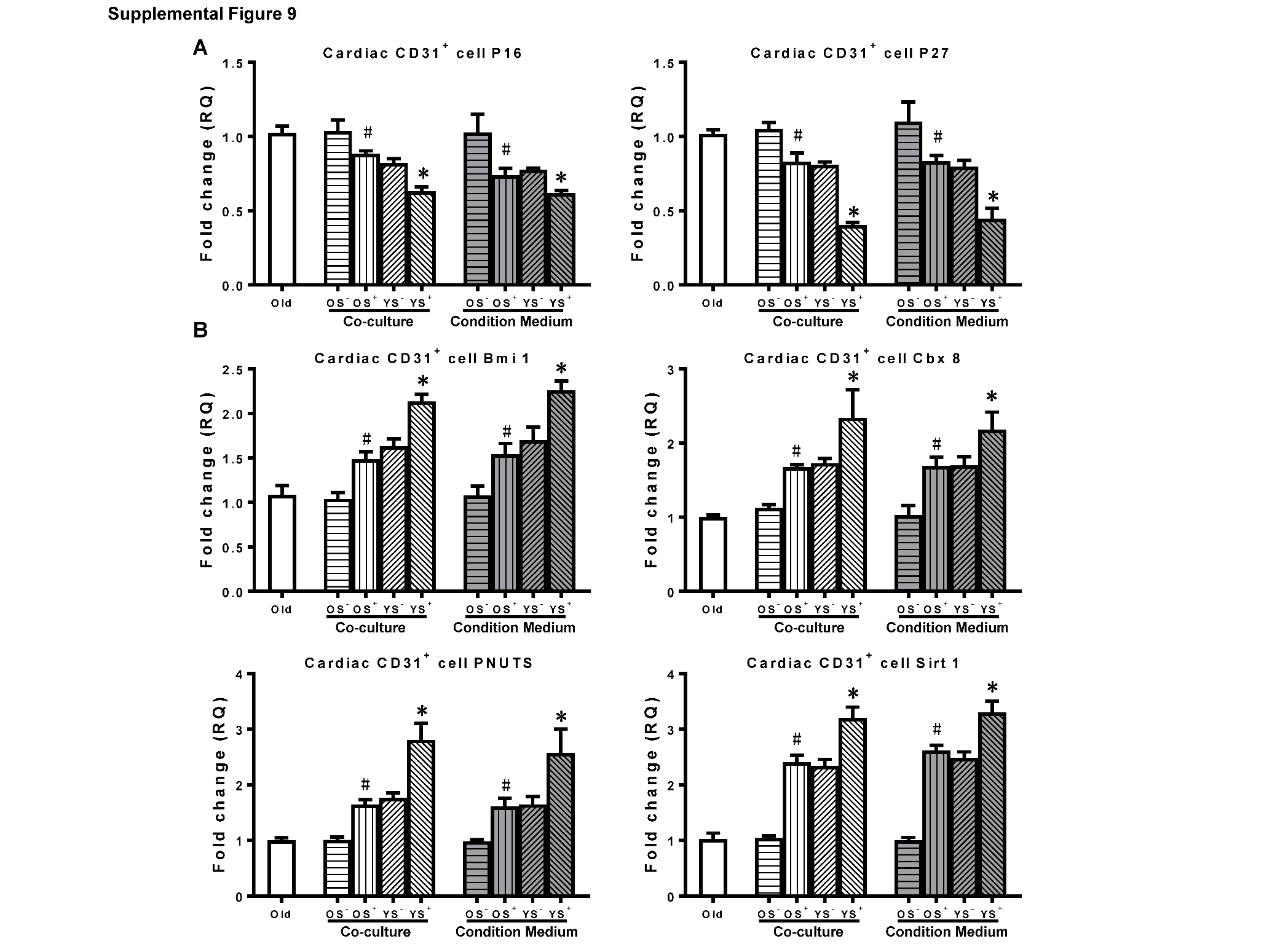
**

**Supplementary Figure 9. The protective effects of BM Sca-1 cells on old cardiac CD31^+^ endothelial cells.**

CD31^+^ endothelial cells were isolated from old (O, 22-23 mos) mouse hearts, and co-cultured with young (Y, 2-3 mos) bone marrow (BM) Sca-1^+^ (YS^+^), Y Sca-1^-^ (YS^-^), O Sca-1^+^ (OS^+^), and O Sca-1^-^(OS^-^) BM cells [respectively](javascript:;) or with the BM cell conditioned medium under hypoxia for 72 h. The mRNA expression of p16^INK4a^ and p27^Kip1^ **(A)**, Bmi1, Cbx8, PNUTS and Sirt1 **(B)** was compared in different treatment groups. n= 6/group; **P*<0.05 YS^+^ *vs* Old, YS^-^, OS^+^*,* or OS^-^ ; ^#^*P*<0.05 OS^+^ *vs* Old, YS^+^, or OS^-^.

**
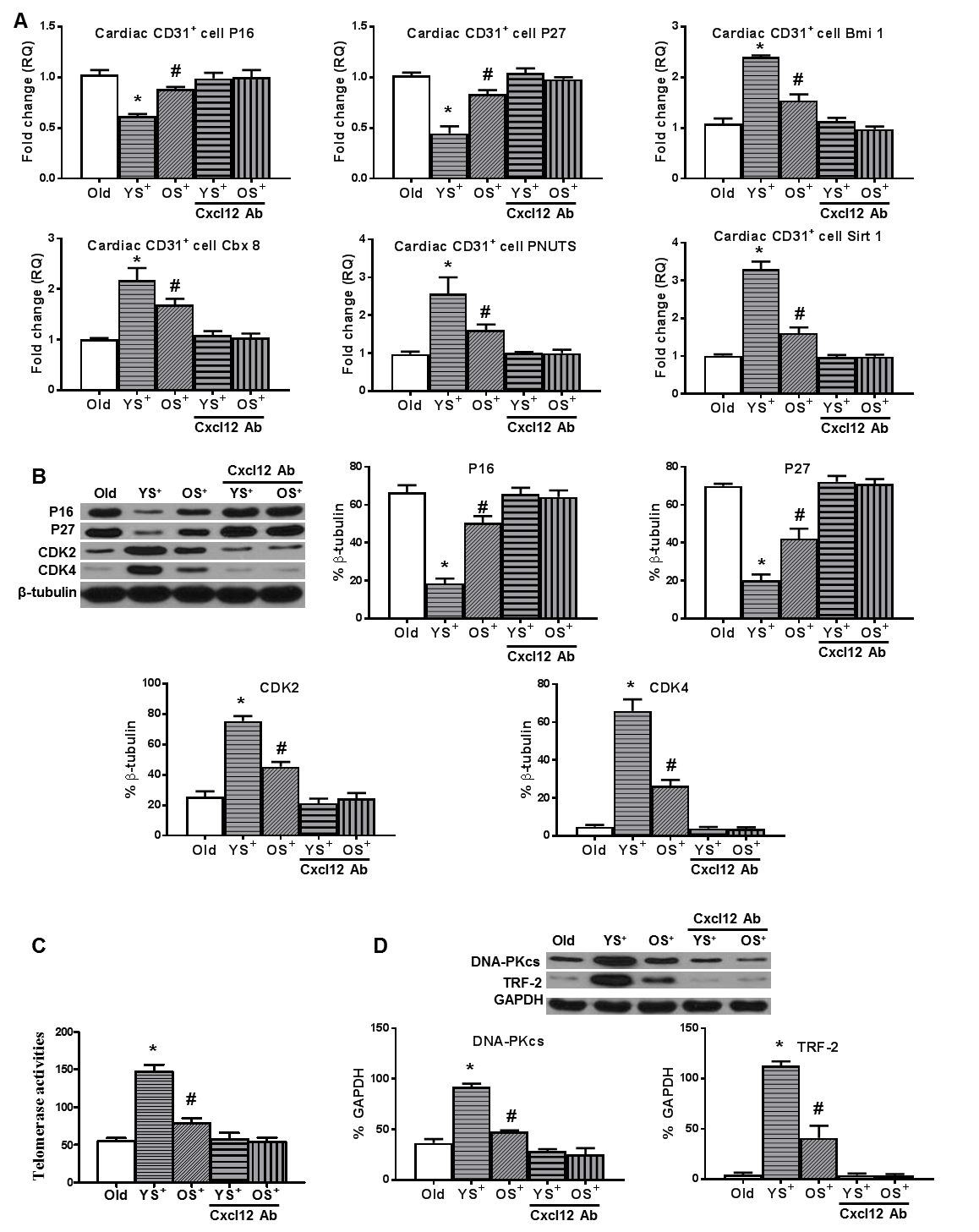
**

**Supplementary Figure 10. The protective effects of BM Sca-1 cells were lost after blocking Cxcl12.**

CD31^+^ endothelial cells were isolated from old (O, 22-23 mos) mouse hearts, and were treated with young (Y, 2-3 mos) and O bone marrow (BM) Sca-1^+^-derived conditioned medium in the absence or presence of a Cxcl12 neutralize antibody (Cxcl12 Ab). **(A**) The mRNA expression of p16^INK4a^, p27^Kip1^, Bmi1, Cbx8, PNUTS and Sirt1 was compared among the different treatment groups. **(B)** The protein expression of p16^INK4a^, p27^Kip1^, CDK2, and CDK4 was compared. **(C)** Telomerase activity and **(D)** telomerase-related protein (DNA-PKcs, TRF-2) expression were compared. n= 6/group; **P*<0.05 YS^+^ *vs* Old, OS^+^, YS^+^+Cxcl12 Ab or OS^+^+Cxcl12 Ab; ^#^*P*<0.05 OS^+^ *vs* Old, YS^+^, YS^+^+Cxcl12 Ab or OS^+^+Cxcl12 Ab.

**
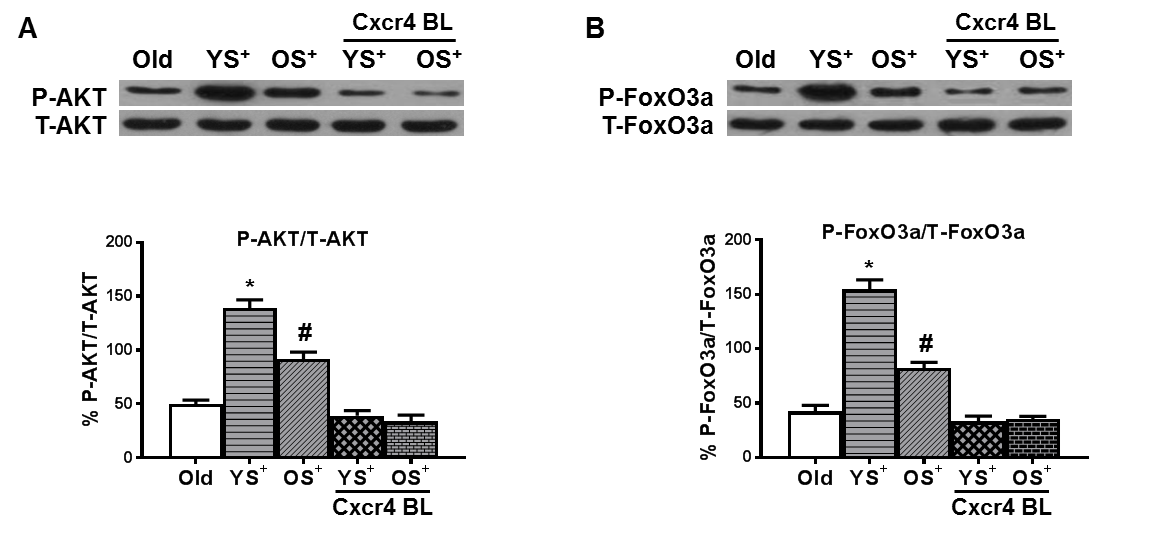
**

**Supplementary Figure 11. The protective effects of BM Sca-1 cells were lost after blocking Cxcr4.**

CD31^+^ endothelial cells were isolated from old (O, 22-23 mos) mouse hearts, and were treated with young (Y, 2-3 mos) or O bone marrow (BM) Sca-1^+^ cell -derived conditioned medium in the absence or presence of a Cxcr4 blocker (Cxcr4 BL). The protein expression of Cxcr4 downstream mediators, total AKT (T-AKT) and phosphorylated AKT (P-AKT), total forkhead box O3a (T-FoxO3a), and phosphorylated FoxO3a (P-FoxO3a), was examined. n= 6/group; **P*<0.05 YS^+^ *vs* Old, OS^+^, YS^+^+Cxcr4 BL or OS^+^+Cxcr4 BL; ^#^*P*<0.05 OS^+^ *vs* Old, YS^+^, YS^+^ Cxcr4 BL or OS^+^+Cxcr4 BL.

**
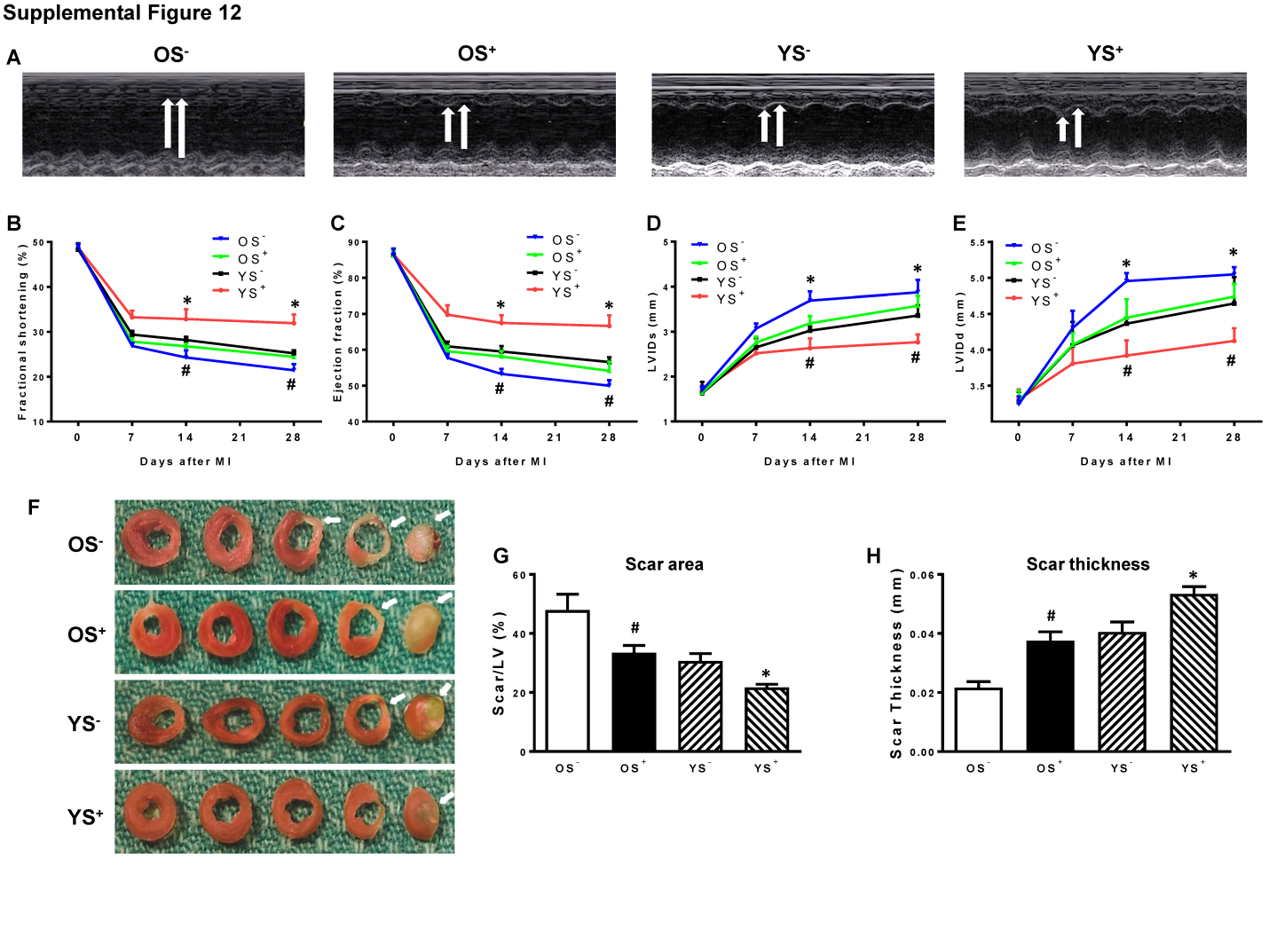
**

**Supplementary Figure 12. BM Sca-1^+^ preserved cardiac function after MI.**

The left anterior coronary artery (LAD) of the 4 chimeric groups was ligated to induce myocardial infarction (MI) at four months after BM reconstitution. Cardiac function was measured by echocardiography at baseline (0), and 7, 14, 21, and 28 days after MI. **(A)** Representative M-mode echocardiographic images. **(B)** Fractional shortening. **(C)** Ejection fraction (EF). **(D)** Left ventricular internal end systolic dimension (LVIDs). **(E)** Left ventricular internal end-diastolic dimension (LVIDs). **(F)** Representative whole sectioned hearts 28 days after MI. Mid-papillary transverse sections depict scar area **(G)** and scar size thickness **(H)**. n=6/group; *P<0.05 YS^+^ *vs* YS^-^, OS^+^*,* or OS^-^; ^#^P<0.05 OS^+^ *vs* OS^-^ or YS^+^.

**Supplementary Tables**

**Supplementary Table 1. Oligonucleotide primer sequences.**

| **Gene name** | **Forward primer** | **Reverse primer** |
| --- | --- | --- |
| P16 | GTGCGATATTTGCGTTCCG | TCTGCTCTTGGGATTGGC |
| P27 | TGGACCAAATGCCTGACTC | GGGAACCGTCTGAAACATTTTC |
| Bmi1 | TTTATGCAGCTCACCCGTC | TTTCCGATCCAATCTGCTCTG |
| Sirt1 | CTCTGAAAGTGAGACCAGTAGC | TGTAGATGAGGCAAAGGTTCC |
| Sirt2 | ACGATGGGCTGGATGAAAG | TTTACCACGCTCTGACACTG |
| Sirt6 | TTCAGCTAGAACGCATGGG | TCTTACACTTGGGACATTCCTC |
| CBX8 | ATTAAGGAGAGCAACACGGAC | GCCACAGTCACCCAGAAATA |
| PNUTS | CCCATAGACCCCAAAGAACTG | GCACCGACTCACCATCTTTC |
| P19 | CTCTGGCTTTCGTGAACATG | TCGAATCTGCACCGTAGTTG |

**References**

Du, C., Li, D., Lin, Y., & Wu, M. (2004). Differentiation of human nasopharyngeal carcinoma xenografts and repression of telomerase activity induced by arsenic trioxide. *Natl Med J India, 17*(2), 67-70.

Smith, J. B., & Brock, T. A. (1983). Analysis of angiotensin-stimulated sodium transport in cultured smooth muscle cells from rat aorta. *J Cell Physiol, 114*(3), 284-290. doi:10.1002/jcp.1041140306

Wang, Y. S., Li, S. H., Guo, J., Mihic, A., Wu, J., Sun, L., . . . Li, R. K. (2014). Role of miR-145 in cardiac myofibroblast differentiation. *J Mol Cell Cardiol, 66*, 94-105. doi:10.1016/j.yjmcc.2013.08.007

Zhou, Y. Y., Wang, S. Q., Zhu, W. Z., Chruscinski, A., Kobilka, B. K., Ziman, B., . . . Xiao, R. P. (2000). Culture and adenoviral infection of adult mouse cardiac myocytes: methods for cellular genetic physiology. *Am J Physiol Heart Circ Physiol, 279*(1), H429-436. doi:10.1152/ajpheart.2000.279.1.H429
